# Identification of Antimotilins, Novel Inhibitors of Helicobacter pylori Flagellar Motility That Inhibit Stomach Colonization in a Mouse Model

**DOI:** 10.1128/mbio.03755-21

**Published:** 2022-03-01

**Authors:** Sebastian Suerbaum, Nina Coombs, Lubna Patel, Dimitri Pscheniza, Katharina Rox, Christine Falk, Achim D. Gruber, Olivia Kershaw, Patrick Chhatwal, Mark Brönstrup, Ursula Bilitewski, Christine Josenhans

**Affiliations:** a Institute for Medical Microbiology and Hospital Epidemiology, Hannover Medical School, Hannover, Germany; b Max von Pettenkofer Institute for Hygiene and Medical Microbiology, Munich, Germany; c Department of Chemical Biology, Helmholtz Centre for Infection Research, Braunschweig, Germany; d Institute of Transplant Immunology, Hannover Medical School, Hannover, Germany; e Institute of Veterinary Pathology, Free University Berlin, Berlin, Germany; f German Center for Infection Research (DZIF), partner site Hannover-Braunschweig, Hannover, Germany; g German Center for Infection Research (DZIF), partner site Munich, Munich, Germany; GSK Vaccines

**Keywords:** *Helicobacter pylori*, drug screening, flagellar motility, motility inhibitor

## Abstract

New treatment options against the widespread cancerogenic gastric pathogen Helicobacter pylori are urgently needed. We describe a novel screening procedure for inhibitors of H. pylori flagellar biosynthesis. The assay is based on a *flaA* flagellin gene-luciferase reporter fusion in H. pylori and was amenable to multi-well screening formats with an excellent Z factor. We screened various compound libraries to identify virulence blockers (“antimotilins”) that inhibit H. pylori motility or the flagellar type III secretion apparatus. We identified compounds that either inhibit both motility and the bacterial viability, or the flagellar system only, without negatively affecting bacterial growth. Novel anti-virulence compounds which suppressed flagellar biosynthesis in H. pylori were active on pure H. pylori cultures *in vitro* and partially suppressed motility directly, reduced flagellin transcript and flagellin protein amounts. We performed a proof-of-principle treatment study in a mouse model of chronic H. pylori infection and demonstrated a significant effect on H. pylori colonization for one antimotilin termed Active2 even as a monotherapy. The diversity of the intestinal microbiota was not significantly affected by Active2. In conclusion, the novel antimotilins active against motility and flagellar assembly bear promise to complement commonly used antibiotic-based combination therapies for treating and eradicating H. pylori infections.

## INTRODUCTION

Helicobacter pylori is a very prevalent gastric pathogen in humans that causes millions of severe gastric disease conditions, including an estimated 900,000 new gastric cancer cases each year worldwide (1, 2). H. pylori eradication is a viable strategy for preventing gastric cancer (3–5). Despite that a general screen-and-treat approach is not recommended, due to the facts that the infection is widespread and that the majority of H. pylori infections persist without causing severe symptoms, there are many ongoing efforts to develop novel treatments and vaccination strategies (6). Disadvantages of the currently recommended treatments to eradicate H. pylori infections are the complex therapeutic regimens, which are always administered as combination therapies, consisting of several different antibiotics, proton pump inhibitors, and potentially other inhibitory compounds, such as bismuth (7–11). These combination therapies, which must be administered for a minimum of 1 week or longer to be effective, frequently lead to severe side effects. In particular a growing concern in recent years, due to a knowledge expansion in this area, are the severe and irreversible effects of common antibiotic-based therapies on the resident human microbiota, predominantly on the microbiome of the digestive tract (12–16). In addition, antibiotic resistance against several currently used antibiotics is easily acquired and on the rise in H. pylori (17–19) and impairs the treatment success of established combination therapies.

H. pylori are capable of directional motility. Motility is conferred by a unipolar bundle of spiral-shaped filamentous motility organelles, the flagella, whose rotation propels the bacteria through the viscous mucus lining of the stomach. A chemotaxis machinery consisting of sensory proteins and signal transducers allows the bacteria to react to chemical and energy gradients by providing either attractant or repellent tactic responses which guide motility (20–23). Directional motility is an essential trait *in vivo* that is required by H. pylori to be able to establish an initial infection and to persist lifelong in the human stomach (21, 24–26). This essential effect during colonization was convincingly demonstrated utilizing motility- or chemotaxis-deficient bacteria in several H. pylori animal model systems, including mice, Mongolian gerbils and gnotobiotic piglets (20, 22, 27–30). Both, the motility and the underlying membrane-inserted nanomachine, the flagellar type III secretion system (31), are therefore attractive targets for the development of novel, possibly supportive therapies against H. pylori, which might be used alone or in combination with other compounds. The flagellar type III secretion system is a very complex multi-protein secretion nanomachine spanning the inner and outer membranes in Gram-negative bacteria, and its assembly is governed by an intricate regulatory hierarchy (31, 32). Apart from providing potential structural targets for novel treatments, the bacterial flagellar apparatus is under tight temporal control by various regulatory mechanisms. In H. pylori, the main known regulatory mechanisms involved in motility and flagellar assembly are transcription and assembly factors (33–35), transcriptional enhancers or repressors (20, 34), small RNAs (36), DNA methylation (37, 38), and global genomic DNA topology (39). The latter two mechanisms probably act in combination with the above-mentioned transcriptional regulators and other DNA binding and bending proteins. Taken together, a plethora of potential targets for inhibitory compounds are present in conjunction with and within the flagellar system.

This attractive situation prompted us to attempt to develop a novel screening tool, based on a flagellar luciferase reporter strain, in order to identify novel inhibitory substances targeting flagellar functions and regulation in H. pylori.

In the present study, we established this tool and used it to screen approximately 4,000 small compounds from various compound libraries. The novel screening procedure helped to identify compounds with direct and indirect activities on flagellar regulation and function. A proof-of-principle treatment study in an H. pylori mouse model confirmed that a non-bactericidal anti-motility compound identified using this strategy was able to exhibit significant *in vivo* activity to reduce bacterial counts in a persistent H. pylori infection. *In vivo*-active anti-virulence compound Active2, while significantly reducing H. pylori colonization, did not affect mouse intestinal microbiota diversity and richness *in vivo*. Hence, the strategy seems to bear promise to identify H. pylori-specific *in vivo* antibacterial agents with less resistance development and fewer side effects, for example on the gastrointestinal microbiota.

## RESULTS

### Library screening identifies numerous compounds active on a H. pylori flagellar assembly reporter strain.

**FIG 1:**
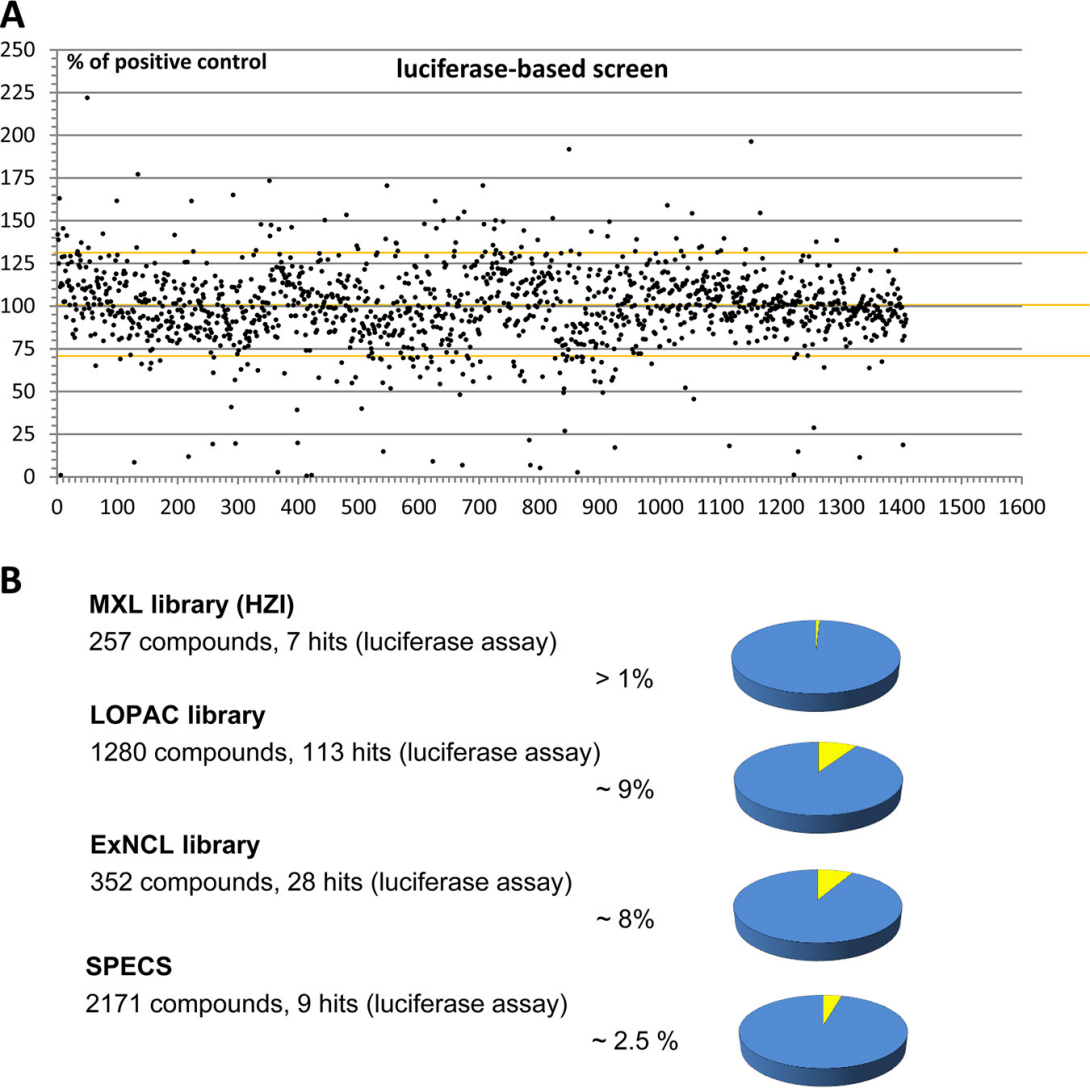
Developing a screening system by primary luciferase-based reporter screen for flagellar biosynthesis of H. pylori in multi-well plates and identification of active small compounds by the screen. A) Results of luciferase flagellar reporter screen for one library of more than 1200 compounds (LOPAC library, library 2); each dot represents one compound result. The *x* axis shows the number of tested compounds, the *y* axis shows reporter activity in percentage of a positive noninhibited control which was set to 100%. Inhibitory as well as enhancing activities of the compounds were observed. Yellow markings depict the borders of the Z factor, corresponding to the 3-fold standard error of the positive (noninhibited) controls. Dots outside the Z-factor margins are considered inhibitory (hit compounds) or enhancing effects. B) Identification of active inhibitory compounds in small compound libraries (see also Table 1) using the primary flagellar luciferase reporter screen. An upper cut-off of 70% inhibition was used to identify and enumerate the strongly inhibitory actives in the primary screen. Active compounds are given in percentage of total screened compounds in each library.

In order to identify small molecule inhibitors of H. pylori flagellar biogenesis, we established a novel screening assay. The core component of this assay was an H. pylori flagellar biogenesis reporter strain that contains a bacterial luciferase gene fused to the late flagellar *flaA* promoter of H. pylori (Methods). This reporter construct was capable of detecting effects on multiple different pathways affecting flagellar gene regulation, assembly and substrate secretion (33, 34), which converge on the inhibition of flagellar motility, which is essential for the organism *in vivo* (20). The screening assay based on the reporter construct was verified to possess a high sensitivity, high signal-to-noise ratio (low background) of 10,000 to 60,000 (determined in relative luminescence units [RLUs]), with a background value of 5 to 10 RLU, and a Z-factor of between 0.6 to 0.8. The positive controls of DMSO-only bacteria performed in eight replicates per plate were always not inhibited (R^2^>95%). The high sensitivity and multi-well format make the assay readily amenable to medium- to high-throughput screening (HTS) (Methods). We subsequently tested about 4,000 compounds from four small-compound libraries (Table 1) in a 96-well format in this assay, revealing compound effects targeting flagellar biosynthesis in H. pylori. This screen identified numerous compounds that exhibited a significant effect on the reporter strain. Actives included both known and novel, yet uncharacterized compounds (Table S1; Fig. 1A). While most active compounds showed inhibitory activity, other compounds increased the activity of the reporter construct. A counter-screen for growth inhibition based on bacterial viability and respiratory activity was performed in multi-well plates, showing that the screen also detected a large number of compounds that had growth-inhibitory effects in addition to the initially detected anti-flagellar effects. The counter-screen also identified compounds that showed only inhibitory effects in the anti-flagellar screen and did not inhibit bacterial growth. Selected compounds with antibacterial activity (including known antibiotics, Ampicillin, Rifampicin, Linezolide) did not show any effects in the anti-flagellar screen, which verified that the screen is not merely detecting antibacterial efficacy in general, but is detecting more specific, flagella-related cellular effects. Overall, about 6% of total screened compounds showed inhibitory activity in the primary flagellar luminescence reporter screen, inhibiting the luciferase output of the assay by more than 70% of the positive control (Fig. 1B). Of all of those primary small-compound actives, only about 1% had inhibitory effects exclusively in the reporter luciferase activity but did not show a direct antibacterial effect.

**TABLE 1:** Small compound libraries used in primary H. pylori luciferase screens

Library	No. of compounds in library	No. (fraction in %) of hit compounds (primary screen, luciferase reduction by ≥ 70%)	Further characteristics of library
MXL	259	39 (15 %); rather weak effects	Natural compound library from Myxobacteria (Helmholtz Centre for Infection Research, HIPS)
LOPAC	1280	113 (8.8 %)	Repurposing compound library, Sigma-Aldrich (https://www.sigmaaldrich.com/life-science/cell-biology/bioactive-small-molecules/lopac1280-navigator.html)
ExNCL	352	28 (8 %)	Natural compound library (University of Tuebingen, Germany)
SPECS	2170	56 (2.6 %)	Commercial compounds, partially enriched for low logD and low molecular mass; SPECS, Zoetermeer, the Netherlands (https://www.specs.net/)

10.1128/mbio.03755-21.6 TABLE S1 Specific compound characterization of selected compounds from LOPAC (repurposing) library with primary hit characteristics on the H. pylori *flaA* luciferase reporter strain. Selected compounds were tested in detail on two different H. pylori strains (N6, P12), demonstrating strain-independent reproducible compound effects. Since most of the compounds had strong effects on the vitality of H. pylori, P12 strain was exclusively tested for MIC and MBC values, revealing similar compund effects on both strains. P12 was slightly more sensitive than N6 in those assays. Bacteriostatic compounds in contrast to bactericidal compounds are distinguishable by high MBC values. The only compound with very little bacteriostatic or bactericidal effect, but strong motility inhibitory effect in this assay panel was flupirtinmaleate. All other compounds had strong antibacterial (“killing“) effects *in vitro*. Download Table S1, PDF file, 0.1 MB. Copyright © 2022 Suerbaum et al. 2022 Suerbaum et al. https://creativecommons.org/licenses/by/4.0/ This content is distributed under the terms of the Creative Commons Attribution 4.0 International license.

### Numerous active compounds have combined anti-flagellar and anti-viability effects on H. pylori *in vitro*.

**FIG 2:**
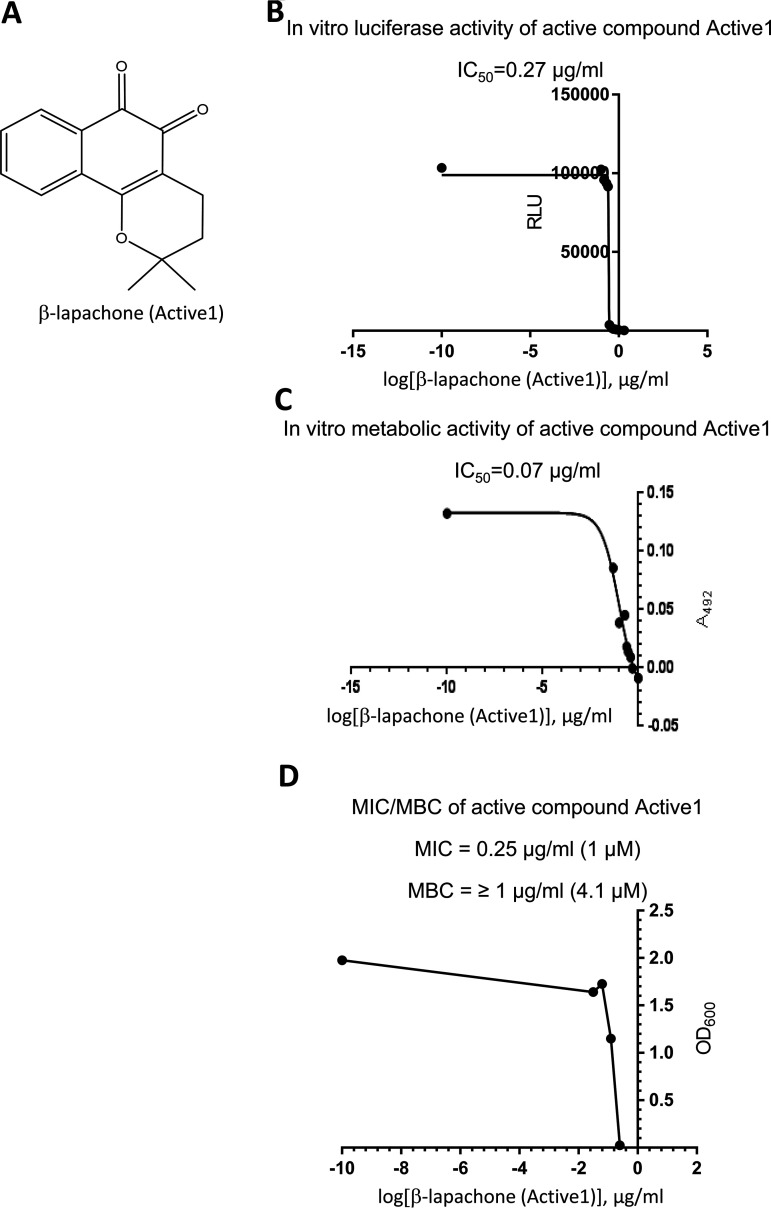
Chemical and antibacterial characteristics on H. pylori of the primary screen hit Active1 (β-lapachone) which had direct specific antibacterial effects against H. pylori. panel A) depicts the chemical structure of Active1 (β-lapachone), which inhibited both flagellar reporter activity and bacterial growth and vitality. In panel B) IC_50_ determination of the *flaA-*luciferase reporter activity for this compound (primary screen), in C) IC_50_ determination for the metabolic activity (counterscreen and assay) and in D) MIC/MBC values for the same compound, which exhibits very strong antibacterial activity against H. pylori, are given.

**FIG 3:**
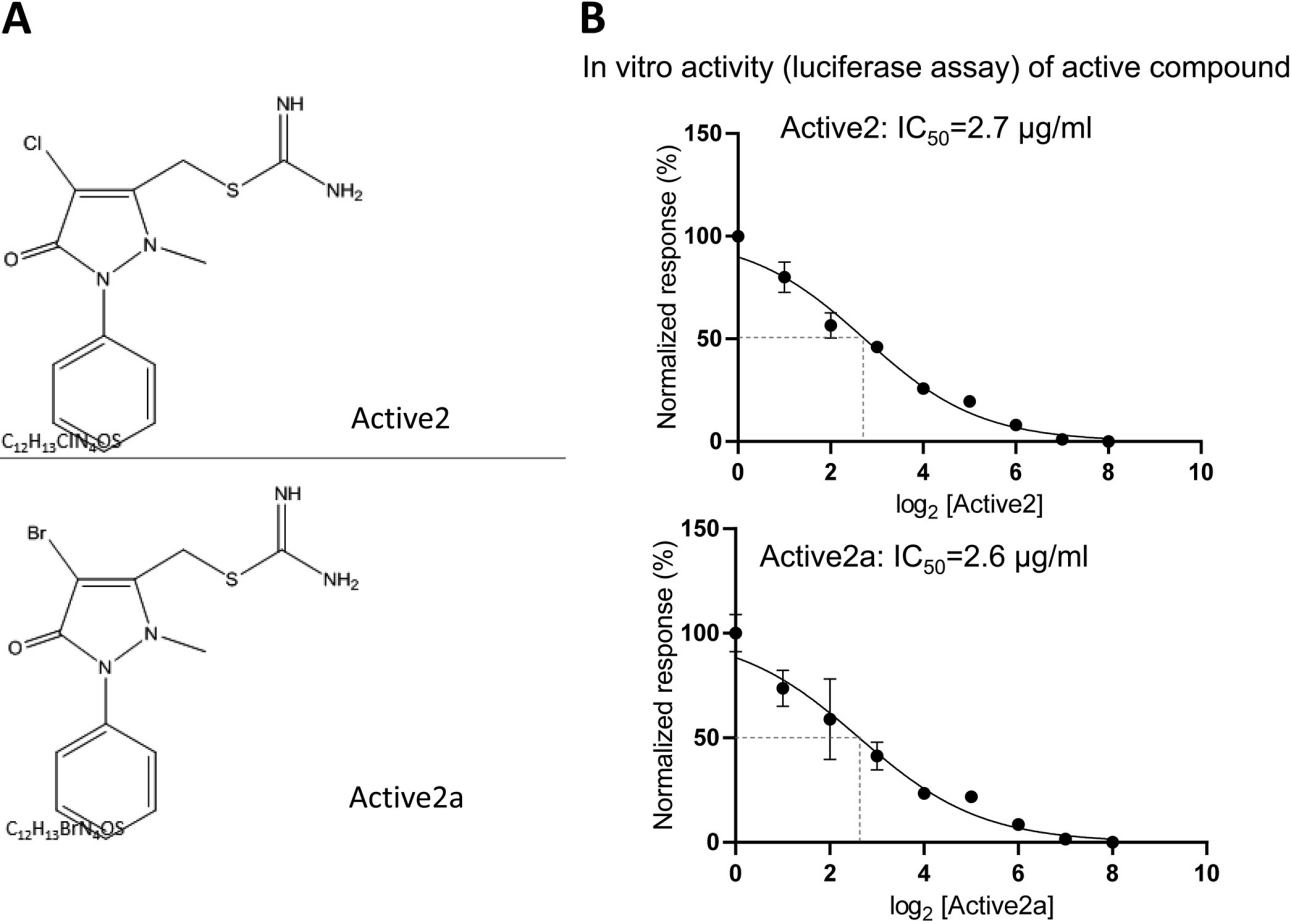
Chemical structure and antiflagellar activity of primary screen active Active2 against H. pylori. The compound was a strong inhibitor in the primary anti-flagellar reporter screen but did not inhibit growth or metabolic activity of H. pylori (in several metabolic assays or in detailed growth inhibition analyses; see also Table 2). Panel A) depicts the chemical structure of the Active2 (top panel) and the close chemical analog Active2a (bottom panel) with similar characteristics; Panel B shows the IC_50_ values for Active2 and Active2a in the primary luciferase-based H. pylori reporter assay.

One drawback to developing a consistent workflow after the identification of compounds using library screening was the restricted availability of most of the compounds, just sufficient for primary screening purposes, and also determines the preliminary quality of the initial screening results. Therefore, for the next steps, we focused on the repurposing library (library 2), for which some of the compounds can be purchased in larger quantities. As already stated above, the majority of all active compounds identified in the primary luciferase screens (on average 80% of all primary reporter actives in library 2) appeared to have also an antibacterial/antiviability effect on H. pylori (DZIF collaborations, personal communication), which can possibly be explained by metabolic effects of those compounds. In order to verify those preliminary results with a smaller set of compounds and quantitate the inhibitory effects of selected compounds in a more definitive manner, we chose a subpanel of 15 commercially available compounds from the repurposing library, library 2, that had shown high activity (≥ 90% reduction of control luminescence activity) in the primary luciferase inhibitory screen. We next determined IC_50_ values for both, flagellar reporter luminescence and bacterial viability inhibition (Fig. 1A). The inhibitory quality of the primary screen results was completely confirmed for all compounds in the subpanel by more detailed testing. Different actives showed a wide range of IC_50_ values (Table S1, Table 2, Fig. 2, Fig. 3), suggesting different levels of activity or diverging activity modes. Therefore, we also determined MIC/MBC values for the selected panel of strongly inhibitory compounds collected in the screening approaches from the repurposing library that contained known therapeutically active compound classes, but excluded known antibiotics. This methodology confirmed that all but one of the preselected active inhibitory compounds exerted a bactericidal or bacteriostatic effect on H. pylori at various concentrations (Table S2). Several compounds were determined to exhibit quite low MIC/MBC values, which highlights them for further study of canonical antibacterial/antibiotic efficacy on H. pylori.

**TABLE 2:** Results for H. pylori screens (luciferase and metabolic assay) and detailed assays of analogous hit compounds Active2 and Active2a; TABLE includes MIC, minimal bactericidal concentration (MBC), and IC_50_ results of luciferase flagellar (*flaA*) reporter assay for both compounds

Compound name	Luciferase screen (% of PC^*a*^)	Metabolic activity (% PC^*a*^)	MIC	MBC	IC_50__luciferase
Active2	1.43	107.7	>32 μg/mL	>32 μg/mL	≤3,05 μg/mL
Active2a	2.59	102.2	>32 μg/mL	>32 μg/mL	≤2,74 μg/mL

a PC = positive control in screen.

10.1128/mbio.03755-21.7 TABLE S2 Specific compound characterization of selected compounds from the LOPAC (repurposing) library with identified primary effects (all antibacterial effects except for flupirtinmaleate) on H. pylori were also tested for effects on C. jejuni strain 11168 and E. coli strain RP437. Some compounds also showed slight antibacterial effects on C. jejuni, indicating similar antibacterial targets in the two more closely related bacterial species. None of the compounds showed a clear antibacterial effect on E. coli. Those assays demonstrated that the novel (antibacterial) compound effects detected in this library were rather specific for H. pylori and possibly other *epsilonproteobacteria*. Download Table S2, PDF file, 0.1 MB. Copyright © 2022 Suerbaum et al. 2022 Suerbaum et al. https://creativecommons.org/licenses/by/4.0/ This content is distributed under the terms of the Creative Commons Attribution 4.0 International license.

### H. pylori-specific effects of anti-flagellar compounds.

One of our present intentions was to single out active compounds which more specifically target H. pylori, instead of developing broad-spectrum antibacterials. Due to the established diversity between various different H. pylori strains, we first wanted to verify, whether compound effects can be strain-specific, which would be a nondesirable trait. For those purposes, we again tested the subpanel of 15 primary active compounds from the repurposing library, using a second, well-characterized H. pylori strain, P12, that differs considerably in its genomic make-up (40). We confirmed that indeed all of the prior effects of the active compounds on H. pylori were not strain-specific. The quantitative antibacterial compound effects (MIC/MBC) were similar for both strains (Table S1). We then tested the same compound panel for their antibacterial activity against two other Gram-negative bacterial species, C. jejuni and E. coli (Table S2). Gut pathogenic C. jejuni was selected, since it has a relatively close taxonomic relatedness to H. pylori, within the Epsilonproteobacteria taxon, while it populates a different habitat and possesses a broader metabolic capacity. E. coli was selected, since it can colonize the gastrointestinal tract, but is a gamma-proteobacterium not closely related to H. pylori. We used MIC/MBC determination, since equivalent reporter strains for the other species were not available and most of those compounds had a strong antibacterial effect on H. pylori, suggesting a primary activity on metabolism. While we obtained weak antibacterial effects on C. jejuni with seven of the selected compounds, only one tested compound had a slight antibacterial effect on E. coli (Table S2). We can therefore conclude that the selected compounds identified as primary actives in the H. pylori screens tended to identify active compounds that rather selectively inhibit H. pylori and did not strongly inhibit two other important Gram-negative GI pathogenic species.

### Identification of anti-flagellar compounds against H. pylori which do not have anti-viability effects and characterization of active pathoblocker compounds for further investigations.

**FIG 4:**
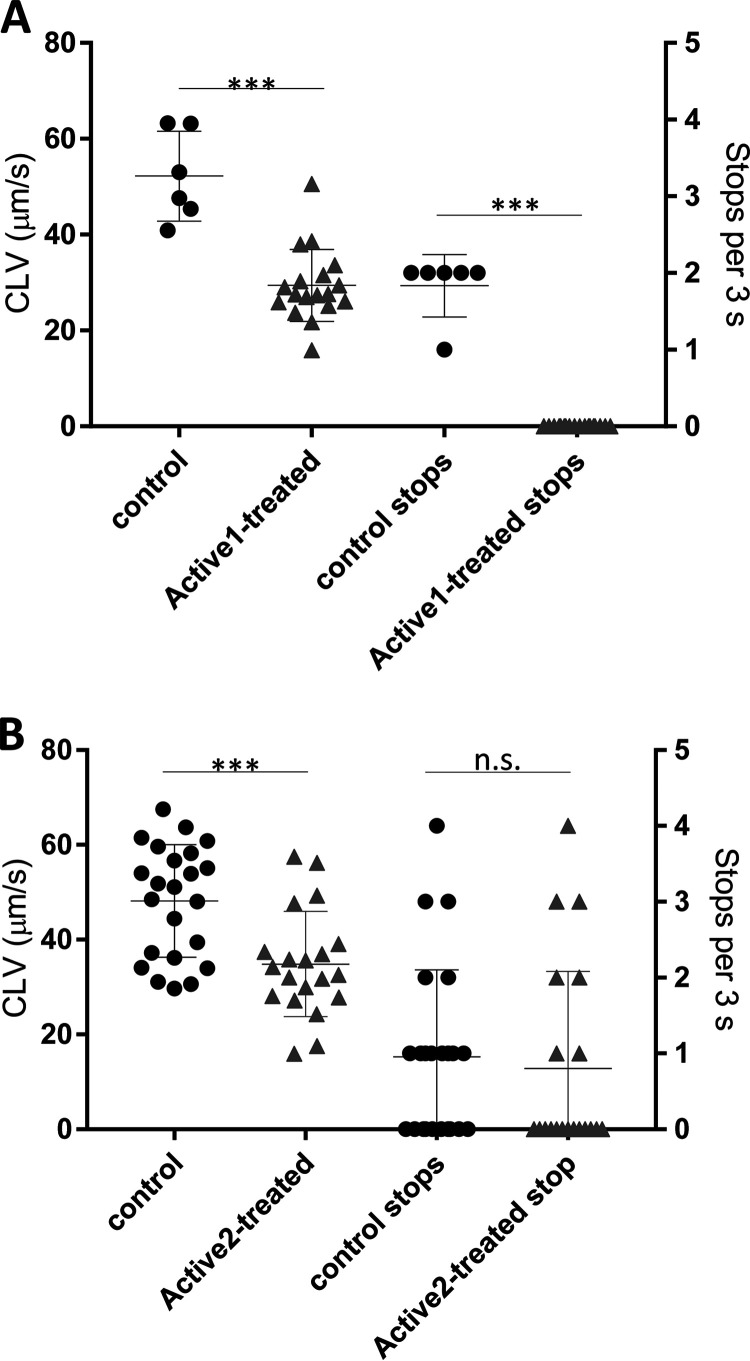
Anti-motility activity of two hit compounds on H. pylori bacteria *in vitro*. Tracking assays with H. pylori were performed in liquid medium (RPMI 1640 with 3% horse serum) and movies recorded in a CELL-R live imaging system (Olympus, see methods). In (A) and (B) curvilinear velocity (CLV) and Stops/reversals for bacteria exposed to Active1 (A) and Active 2 (B) at a concentration of 10 μg/mL during the swimming runs of the bacteria (at least 20 cells recorded), as a measure of intact taxis and directional motility were quantitated as described in (74). Significance of differences between the positive control (Control) and the compound-treated bacterial samples are indicated by asterisks. *** <0.001. n.s. = nonsignificant difference.

Our primary purpose was to identify compounds that exhibited anti-flagellar but not anti-viability effects. We identified some compounds (on average 1% of all primary actives) from the libraries which had a primary inhibitory effect on the flagellin-luciferase reporter, but appeared not to have antibacterial activity, which singled those out for further studies on their specific anti-flagellar effects. As an important approach to assess one potential mechanism of the anti-vital and anti-flagellar effects, we tested again a panel of 10 active compounds (Library 2) of which sufficient amounts were available, on H. pylori gene regulation using semiquantitative or quantitative RT-PCR (qPCR). Effects found for most of those primary active compounds which were clear antibacterials also included a strong reduction of various important H. pylori transcripts (flagellar as well as stress- and metabolism-related; Fig. S1A), underlining their general killing activity. Indeed, very few compounds identified in the primary screen had no antibacterial efficacy as assessed by either a metabolic test for the activity of the respiratory chain, ATP (BacTiter Glo) content assay, or directly by MIC growth inhibition assay. We thereby singled out a few active compounds which appeared either to modulate only luminescence activity in the flagellar screen, or which seemed to target both, motility and metabolic targets, as verified by secondary antibacterial screens or tests (Table 2, Fig. 2, Fig. 3). Selected compounds out of these actives were further tested in direct motility assays on H. pylori. Two compounds, Active1 and Active2, exhibited a strong activity against flagella-associated functions in the primary screen and might represent active patho-blocker compounds. Active1, which also had very strong antibacterial effects on H. pylori, belonged to the family of β-lapachones, which have been described as canonical strong antibacterial natural compounds from Lapacho tree bark active on various bacterial species and parasites long ago (41–46). One validated cellular (metabolic) target of β-lapachone is the NAD(P)H:quinone oxidoreductase-1 (NQO1) subunit of membrane respiratory complex I (47), which has led to the proposition of using lapachones in a variety of indications, including the treatment of tumors, parasites, fungi or bacterial infections, as its target is ubiquitous and essential in bacteria (46). We performed direct motility (taxis) assays with Active1, which strongly inhibited motility and targeted motility at low concentrations after short exposure times (Fig. 4). Due to its poly pharmacological, antivital (Fig. 2) properties and poor solubility, β-lapachone was not characterized further at this stage. The second selected compound, Active2, and its close relative Active2a, are phenyl-pyrazolones (Fig. 3), without any prior known biological effects on any molecular target or on bacteria. They possessed strong activity in the H. pylori antimotility assays, as indicated by IC_50_ values of 2.7 μg/mL and 2.6 μg/mL, respectively (Fig. 3), but did not exhibit any canonical antibacterial effect (MIC > 32 μg/mL), which made them particularly interesting for our further assays. Both compounds displayed good water solubility. We selected the chlorinated analog Active2 for detailed motility analysis and found that it reduced motility speed after a short time of co-incubation at physiological concentrations (10 μg/mL) (Fig. 4). This compound was also active with a similar inhibitory effect in another H. pylori strain, L7, which we also engineered to express the same *flaA*-luciferase reporter fusion as the construct chromosomally inserted in strain N6. In addition, we also tested in total four H. pylori isolates, three clinical and one animal-adapted (strains N6, L7, P12, HP87 [24]) for *flaA* reporter or transcript reduction in short-term exposure experiments to Active2, using RNA isolation and qPCR quantification (Methods). These experiments confirmed a reduction of *flaA* transcript in the tested strains in comparison to a non-exposed sample (Fig. S1 B). We also detected a reduction of main flagellin A protein by Western blotting under the influence of Active2, in particular in the surface-exposed bacterial fraction (Fig. S2).

10.1128/mbio.03755-21.2 FIG S1 Regulatory activity of primary small compound actives on specific and transcript amounts (RT-PCR). A) depicts semiquantitative (sq) RT-PCR results from RNA isolated from H. pylori N6 treated for 1 h in liquid culture of mid-log-phase with 10 μg/mL of selected compounds (dissolved in DMSO) from the LOPAC library, containing repurposing compounds. Numbering of compounds in A) 1 -Calmidazolium, 2 -b-Lapachone (Active1), 5 –L745,870, 6 –NSC95397, 9 -paraquat, 10 –L-750,667, 11 –Tyrphostin AG879, 20 -ZPCK, 21 –SP600125, 22 –Bay11-7985; compounds are also listed in Tables S1 and S2; compound 2 in A) is Active1= β-lapachone. Strongly active compounds are boxed in red color, which reduced transcript amounts of class 2 (*flgS*, *fliA, yellow*) and class 3 (*flaA, yellow*) flagellar genes as well as stress related (*arsS, green box*) and metabolic (*nqo13, violet box*) genes. In this case, the compounds had canonical antibacterial effects (see also Table S1 for MIC/MBC values). c are sq RT-PCRs performed on non-treated H. pylori N6 bacterial controls (DMSO only) incubated under the same conditions. Paraquat (9, not included in compound library) was used as an examplary set-up for a compound introducing oxidative stress. Gene abbreviations for RT-PCT: *cagC*, functionally important gene of the *cag* pathogenicity island; *flaA,* flagellin A subunit; *flgS*, flagellar regulator two-component system (TCS), sensory protein; *fliA*, flagellar sigma factor σ28; *arsS* sensory domain of acid sensing TCS; *groEL*, heat shock protein, large subunit; *trxR1*, thioredoxin reductase; *porA*, pyruvate-ferredoxin oxidoreductase subunit; *ccoN*, N subunit of Cbb3 terminal oxidase; *nqo13*, subunit of complex I of respiratory chain; (B) quantitative RT-PCR (qRT-PCR) results from RNAs collected from Active2 (BL2) treatment in vitro (at 20 μg/mL for 4 h in liquid culture) of H. pylori mouse-adapted strain HP87 (wild type strain) and HP87 bacteria reisolated from BL2-exposed mice from the treatment study (38A: mouse 38, antrum; 38C: mouse 38 corpus; 39C: mouse 39 corpus; mouse identities and group association see also Table 3 and Fig. S3), and of independent H. pylori wild type strain P12. Strain N6 has been characterized for Active2-effects on *flaA* by reporter assays. The HP87 reisolates from treated mice showed a similar response to Active2, indicating no primary acquired resistance to the compound effect on *flaA* during the treatment study. All *flaA* transcript amounts were normalized to 16S transcript. In this experiment under the short-term influence of Active2, no other gene transcripts of the above gene panel in A) were changed or downmodulated in the bacteria by Active2, coinciding with a lack of immediate growth- or stress-related Active2 effects on the bacteria, observed also in other assays for Active2. Download FIG S1, PDF file, 0.6 MB. Copyright © 2022 Suerbaum et al. 2022 Suerbaum et al. https://creativecommons.org/licenses/by/4.0/ This content is distributed under the terms of the Creative Commons Attribution 4.0 International license.

10.1128/mbio.03755-21.3 FIG S2 Western blot reveals reduction of flagellin protein FlaA upon incubation with active antimotilin compound Active2. Two different time points of an H. pylori growth curve in liquid BHI medium (3% yeast extract, 5% horse serum), T1 (early exponential, OD_600_ of about 0.4), and T2 (late exponential growth, OD_600_ of about 1.5) were monitored for differences in protein expression in the continuous presence (+) or absence of Active2 compound. Western immunoblots were performed on the insoluble (IS), soluble (S) and surface-associated (OF) fractions of the bacteria. Blots were developed with anti-FlaA, anti-FlhA and anti-CagL antisera as indicated. The FlaA blot reveals a significant reduction of main flagellin FlaA at time point T2 upon supplementation with Active2 compound, both in the soluble and surface-associated bacterial fractions (red arrows). FlhA serves as a fractionation control for the insoluble fraction. CagL, which was slightly increased by Active2 compound at both time points, is shown as a second control for the insoluble fraction. Red marker band of prestained molecular mass marker runs at about 54 kDa. Download FIG S2, PDF file, 0.2 MB. Copyright © 2022 Suerbaum et al. 2022 Suerbaum et al. https://creativecommons.org/licenses/by/4.0/ This content is distributed under the terms of the Creative Commons Attribution 4.0 International license.

### Active2 pathoblocker compound reduces colonization in a H. pylori mouse model.

**FIG 5:**
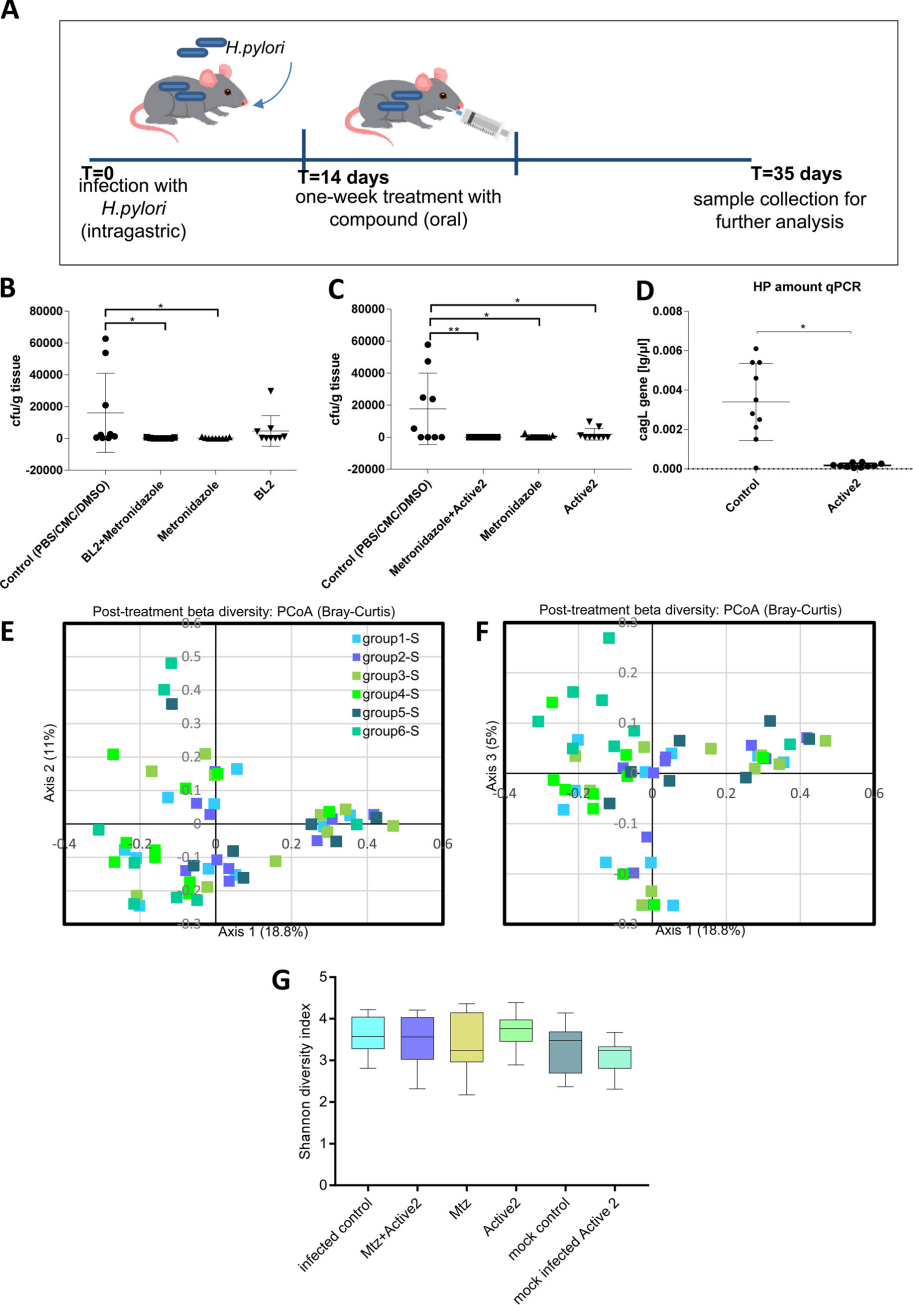
Proof-of-principle therapeutic application of a primary active patho-blocker antimotilin compound (Active2/BL2) in a chronic H. pylori mouse infection model revealed significant activity *in vivo*. (A) a scheme of the experimental design of the mouse experiment. (B), the CFU values of four groups of H. pylori-infected mice (groups 1, 2, 3, and 4) with or without administration of BL2 (Active2) compound in combination with metronidazole, or after sole administration of BL2 (group 4), are shown for corpus recovery of H. pylori. For mouse group identities, see Table 3. (C) the CFU values of H. pylori recovery from the antrum for the same four groups of mice are depicted. (D) the quantitative results of primer-probe qPCR directed against the H. pylori *cagL* gene for all single mice (corpus) of mouse group 1 (infected, not-treated control group) and mouse group 4 (infected and Active2-treated mice). The results are shown as absolute values [pg DNA per μl solution), normalized to individual tissue weight of the biopsy specimen. (E–G) the results of 16S amplicon-based microbiota analysis of all mouse groups (groups 1 through 6, see Table 3) in the therapeutic study. (E–F) Bray-Curtis analysis of microbiota beta-diversity as depicted in principal-component analysis (PCoA charts). Shown are the primar*y* axes 1, 2, and 3, which reflect the highest percentages of microbiota diversity between the single mice. Each symbol represents one mouse sample, with different symbol colours for each group as shown in the graph legend. The variation for each sample described b*y* axis 1 and axis 2 (panel E), or b*y* axis 1 and axis 3 (panel F), respectively, is depicted in the graphs. The microbiota composition (beta-diversity) between groups was not significantly different (multiple pairwise comparisons, AMOVA), as shown in the statistics Table S3 in the Supplements. (G) alpha-diversity between the mouse groups as calculated using Shannon-Wiener diversity index; single groups depicted as box plots with their respective mean and standard deviations; the differences between groups are not significant (One-Way ANOVA, multiple pairwise comparisons). Two groups which were not infected, but only administered Active2 or only mock-dosed, are not included in panels B and C. Statistics in panels B, C, D were performed using pairwise comparisons by One-Way ANOVA; * *P* < 0.01; ** *P* < 0.001. The detailed experimental set-up is listed in the methods.

Active2, exhibiting strong *in vitro* activities on the luciferase assay (IC_50_ of about 2.7 μg/mL) and mediated direct motility inhibition, but did not affect bacterial vitality (MIC > 32 μg/mL), was selected for a proof-of-principle therapeutic experiment in H. pylori-infected mice *in vivo*. After having clarified that the compound is not toxic to mice at doses below 60 mg/kg/day, within the proposed range of a promising therapeutic activity *in vivo*, it was further tested for therapeutic efficacy in a mouse model of chronic H. pylori infection (Fig. 5A).

10.1128/mbio.03755-21.8 TABLE S3 Statistics using AMOVA (Bray-Curtis) of 16S amplicon-based microbiota analyses (beta-diversity) of fecal pellets from Active2-treated mice and controls. The statistical analyses indicate no significant difference of the intestinal commensal microbiota composition (beta-diversity) between groups before or after the experimental course. Animal groups 1 through 6 are numbered as outlined in Table 3; additional group designation a: fecal microbiota before start of experiment after acclimatization period; additional group designation b: fecal microbiota after experimental course. P values of the respective comparisons are shaded in blue (nonsignificantly different) or light orange (trend towards significance). Differences between all mouse groups before the experiments (a) were also not significant; only one pairwise comparison between groups 1a and 6a as one representative comparison is listed for the preexperimental status. Download Table S3, PDF file, 0.7 MB. Copyright © 2022 Suerbaum et al. 2022 Suerbaum et al. https://creativecommons.org/licenses/by/4.0/ This content is distributed under the terms of the Creative Commons Attribution 4.0 International license.

Six groups of mice (10 mice per group in each of the four infected and/or treated groups, and eight mice in both control groups, Table 3) were defined in this preclinical mouse treatment trial. For the treatment groups, mice were infected in week one with a mouse-adapted stable *cagPAI*-positive H. pylori strain, HP87P7, and the chronic infection was manifested for two more weeks. The once-daily treatment (intragastric gavage) started in week four of the trial (Fig. 5A) and was performed on seven consecutive days. For the treatment groups, three H. pylori-infected groups were evaluated in parallel to which the compound was administered orally in sterile cell culture-grade PBS. One dose of treatment was given daily. Group one was infected and mock-treated by administering only PBS. Group three was treated with metronidazole only (14.3 mg/kg/day), an established anti-H. pylori antibiotic (4). The antibiotic dose corresponds to dosing routinely used in human combination treatment. We treated infected group two with metronidazole (dosed at 14.3 mg/kg/day) and Active2 (10 mg/kg/day) in combination, and infected group four received Active2 (10 mg/kg/day) only. In addition, two mock-infected groups were set aside, one for administering Active2 only (10 mg/kg/day; microbiota compound control) and one which was mock-infected and mock-treated (null control for microbiota and immune response). The primary read-out of successful treatment was the reduction of CFUs of bacteria in the stomachs of infected and treated mice, over infected, nontreated, mice, at the end of the experiment. We determined a significant therapeutic activity of Active2 against H. pylori, with a statistically significant reduction of CFU counts in the stomach antrum for the infected Active2-treated group four in comparison to the infected-only control (group one) and a trend toward reduction in the corpus (Fig. 5B and C). As expected, the metronidazole-only group and the combined metronidazole and Active2 treatment group also had significantly reduced live bacterial counts at recovery in comparison to the infected control, since most of the mice in those groups did not exhibit any recovery of live H. pylori (Fig. 5B and C). All mice in the infected, positive-control, group were colonized (both CFU counts and PCR-positive), although the CFU counts in three positive animals were low.

**TABLE 3:** Treatment groups in chronic H. pylori mouse model for small anti-motility compound BL2 (Active2)

Mouse group	No. of animals (n)	HP-infected (Y/-)*	Metronidazole (Y/-)*	Active2 (Y/-)*	Not-treated (Nt)
1	10	Y	-	-	Nt
2	10	Y	Y	Y	
3	10	Y	Y	-	
4	10	Y	-	Y	
5	8	-	-	-	Nt
6	8	-	-	Y	

*Y = yes; - = no.

When we tested the presence of residual H. pylori bacteria in stomach homogenates of CFU-negative mice using H. pylori gene-specific PCR, we found that upon antibiotic/metronidazole treatment, or upon combination treatment using both antibiotic and Active2 compound, all mice still had detectable specific bacterial signal in their stomachs (2 weeks posttreatment; Fig. S3A). When we Sanger-sequenced the specific PCR signal after DNA purification, we obtained correct H. pylori gene sequences (*cagL*; Fig. S3B) for all these PCR-amplified bands. Surprisingly, the infected mouse group that had been given Active2 compound only, in contrast had a strong reduction not only of CFU but also of gene-specific PCR signal at 2 weeks posttreatment in all but two mice, matching low CFU recovery (Fig. S3A). To verify this surprising result and the significantly lower CFU counts in the Active2-treated group, we established a quantitative primer-probe qPCR of gastric tissue, based on the H. pylori *cagL* gene (Methods). When we evaluated gastric tissues of the infected Active2 treatment group against the infected untreated group (and the other infected groups) by qPCR, we obtained significantly lower qPCR counts in the stomach corpus for all mice from the Active2-treated group, with DNA amounts close to the detection limit of five bacterial genome copies in this group (Fig. 5D). In contrast, the qPCR results for all metronidazole–treated mice in groups two and three were not significantly different from the nontreated infected control group one (not shown), in contrast to the severely reduced CFU counts.

10.1128/mbio.03755-21.4 FIG S3 PCR identified strong residual bacterial load in a therapeutic H. pylori chronic infection model for the antibiotic metronidazole but not for the active pathoblocker/antimotilin compound BL2. No significant influence on plasma cytokine levels. (A) a polymerase chain reaction for a partial nucleotide segment of the H. pylori *cagL* gene (Methods) was performed on stomach corpus homogenates from the chronic mouse infection and treatment experiment conducted with BL2. PCR results from mice grouped in four groups (only H. pylori-infected groups shown; groups separated by blue lines) (Table 3) are shown in the order of appearance. Group 1: mice 1 to 10; group 2: mice 11 to 20; group 3: mice 21 to 30; group 4 mice 31 to 40. Mouse numbers are shown above the PCR lanes. High-quality Sanger sequences obtained from *cagL* positive PCRs in the absence of positive culture (except for mouse 38, where two reisolate clones could be recovered) are boxed; respective Sanger sequence reads are shown in B). Nucleotide sequences from mice 29, 36 and 38 contain SNPs in comparison to the input strain (red box), while the sequences from the other reisolates and homogenate PCR bands do not contain SNPs. *n* = negative control without DNA; *P* = positive PCR control using genomic DNA of mouse-adapted H. pylori strain; L = nucleotide band size ladder-1 kb ladder, Thermo Scientific. C) No significant change of plasma cytokine levels (measured by BioRad Bioplex cytokine multiplex assay) was determined in chronic mouse infection model with or without therapeutic intervention with Active2. Groups of mice (six groups, 56 mice) are grouped as depicted in Table 3 and in the results. Results are shown in a cumulative manner in bar graphs, in pg/mL for each cytokine. Colour labels for each cytokine are given in the figure. Of the mock-infected control groups 5 and 6, only every second mouse was tested as indicated. Differences between groups of mice were nonsignificant. Download FIG S3, PDF file, 0.3 MB. Copyright © 2022 Suerbaum et al. 2022 Suerbaum et al. https://creativecommons.org/licenses/by/4.0/ This content is distributed under the terms of the Creative Commons Attribution 4.0 International license.

Histopathology evaluation of all infected mouse groups, with or without treatment, failed to show any elevated stomach pathology in any of the animals, with no statistically significant differences between mean scores of all groups (data not shown). Low stomach histopathology has been observed before with all H. pylori mouse models, due to so far unknown biological causes which generally limit pathology by H. pylori in the mouse. Gastric pathology may also vary in humans and therefore is not a suitable single read-out parameter of successful infection or disease marker. Hence, since the hallmark and endpoint of our model is based on the reduction of bacterial CFU (bacterial eradication) and not on pathological evaluation, our *in vivo* results confirm the effective antibacterial treatment. It is also important to note that administration of Active2 did not lead to a stronger pathology in infected/treated or Active2-only control mice.

### Fecal microbiota composition and plasma cytokines are not significantly altered by a therapeutically effective compound in the H. pylori infection and treatment mouse model.

From the experimental mouse treatment study with compound Active2, we harvested fecal pellets from all groups of mice at the end of the experiment (before necropsy). Hence, we could answer questions concerning the influence of H. pylori infection alone or of therapeutic compound administration on the fecal microbiota composition of all animals. We analyzed fecal microbiota composition upon microbiota 16S rRNA amplicon sequencing of the V3-V4 variable regions. Active compound Active2 alone did not exert a marked influence on fecal microbiota composition (beta diversity) (Fig. 5E and F) or diversity (alpha diversity) (Fig. 5G, Fig. S4). Also, the combination of H. pylori infection and compound administration did not lead to a marked difference in composition or diversity in comparison to the infected or negative-control groups in the treatment study (Fig. 5E; Table S3). Testing mouse plasma from the treatment study for systemic cytokine production in blood by multiplex bead test (Methods) did not reveal any significant changes in cytokine amounts in blood between the control groups, the infected groups and the compound- and/or antibiotic-treated groups (Fig. S3C).

10.1128/mbio.03755-21.5 FIG S4 Microbiota composition in the H. pylori chronic infection model posttreatment. All groups of mice were evaluated using 16S amplicon sequencing (Illumina MiSeq) from fecal pellets and subsequent OTU analysis, which were further grouped into taxonomic units (six groups, 56 mice, group details in Table 3 and in the Methods/Supplemental Methods). Shown here is the grouping into bacterial taxonomic classes, depicted as bar graphs for each mouse. Known classes of bacteria are shown with their respective color-coding in the legend below the figure; ND (orange) signify a small number of OTUs in the samples that could not be grouped into any of the known taxonomic classes. Main identified classes in all mice and mouse groups were the Bacteroidia, Clostridia and Bacilli. Differences in alpha diversity between mouse groups were not significant (Table S3). Download FIG S4, PDF file, 0.1 MB. Copyright © 2022 Suerbaum et al. 2022 Suerbaum et al. https://creativecommons.org/licenses/by/4.0/ This content is distributed under the terms of the Creative Commons Attribution 4.0 International license.

## DISCUSSION

The first antibacterial therapies against H. pylori infection of the human stomach were developed in the 1980s and 1990s, using antibiotics licensed for other applications that displayed *in vitro* activity against the stomach-infecting bacteria. These antibiotics were then rapidly evaluated in clinical trials, either alone or in combinations (7, 10, 48). The most effective antibacterial combination regimens currently achieve eradication rates of > 90% (6). Current treatment regimens have to be administered for one to 2 weeks and are frequently accompanied by side effects. They are hampered by increased resistance development of the bacteria (18) and long-term effects on the microbiota (16, 49). It has been reported that the H. pylori infection in humans cannot readily be eradicated using one single antibiotic, which may be explained by relapsing infection or insufficient local antibiotic concentrations in the gastric mucus (50), or by bacterial retainment in “sanctuary zones” (51, 52), rather than by failure of a primary antibacterial effect. This is the main reason why currently only combination therapies are being used for treating the human infection.

Despite that the population burden of H. pylori infections, associated diseases, and deaths has been reduced in some geographical locations, it is not significantly decreasing on a worldwide scale, remaining at larger than 50% of the world population (53). It is therefore highly desirable to identify novel therapeutic options, designed to treat H. pylori specifically, and possibly with fewer side effects in particular with respect to the microbiota. A vaccine against H. pylori is not clinically available (54–56).

Recently, several novel therapeutic agents, mainly with canonical antibiotic effects, have been tested for efficacy in H. pylori mouse models. Gavrish and colleagues (57) have identified a novel antibacterial compound, HPi1, of yet unknown target specificity. Two other promising compound classes have been recently developed in the H. pylori field: Amixicile, a nitazoxanide (amino-nitrothiazole amide) compound, belongs to a classical antibacterial compound class and targets the bacterial H. pylori PFOR enzyme (58). Nitazoxanide itself was also identified in our primary and secondary screens to exert strong antibacterial activity against H. pylori, but this compound does not work effectively as an antibacterial treatment *in vivo* in mouse models or humans (59, 60). The other compound class recently pursued as patho-blocker therapeutics against H. pylori are the urease inhibitors, for example of the aryl-amino hydroxamate class, which were active in short-term treatment experiments against H. pylori in a mouse model (61). The compounds do not kill the bacteria, but target bacterial urease, an enzyme long known to be absolutely essential for H. pylori survival in the stomach *in vivo* (30, 62, 63). Similarly, a very recent patho-blocker approach tested *in vitro* targets H. pylori carbonic anhydrase, another important metabolic enzyme *in vivo* which is not essential *in vitro* (64). All types of novel inhibitors recently tested *in vitro* or in animal models are more specific against H. pylori than currently used therapies based on broad-spectrum antibiotic combinations. Hence, it is visible that multiple targeted efforts are under way to move from broad-spectrum therapies into selected therapies against H. pylori. In general, patho-blockers specifically acting against various different bacterial pathogens are being developed against biofilm formation, adherence, motility or bacterial toxins (65–67). Despite being timely, specific patho-blocker approaches have not been widely evaluated against H. pylori, and the development of a successful screen in this area was, to our knowledge, not yet published or broadly discussed. Hence, in the present study, we have centered our efforts on the development of a well-applicable screen to identify novel compounds, which act specifically on H. pylori to block its flagellar gene regulation, motility, chemotaxis, or flagellar assembly. Others have recently, in parallel with our study, endorsed the general concept of anti-motility therapies against various human pathogens (68). The concept of motility inhibition as novel, specific therapy was already evaluated *in vitro* against the intestinal diarrheal pathogen C. jejuni (69) but identified only compounds with a classical antibacterial activity (70). Another anti-virulence approach has directly targeted the sodium-propelled flagellar motors of Vibrio cholerae (71), and identified such inhibitors *in vitro*, which also had weak antibacterial effects.

We have developed a powerful luciferase-based high-throughput screening system to identify broad antiflagellar and antimotility effects against H. pylori. Using this screening approach, we identified numerous active compounds with both, strong anti-bacterial and/or anti-flagellar effects on H. pylori. While many of the classified active compounds primarily exhibited antibacterial activities, we were also able to identify compounds that had anti-flagellar effects without suppressing bacterial growth or viability. Several primary actives had low antibacterial MIC values and acted on H. pylori probably via metabolic inhibition. For further studies, we selected one small molecule, named Active1 (β-lapachone, a 2,4-naphthoquinone) long-known to have antibacterial effects on a variety of species (44, 46), but not yet characterized to be active against H. pylori. β-lapachone derivatives, due to their cell-killing activity, are currently being further developed as anticancer agents (45, 47, 72) and were also strongly active against H. pylori in our hands. Furthermore, we selected the phenyl-pyrazolone small compound Active2, which represents a novel class of anti-virulence, anti-motility compounds with a yet unknown target, for further studies of its activities against H. pylori. Active2 was rather specifically active in H. pylori (no antibacterial activity on E. coli, C. jejuni, P. aeruginosa, S. aureus; no antimotility activity on C. jejuni) which also led us to prioritize it for a first proof-of-principle therapeutic study in an H. pylori mouse infection model.

The therapeutic application of the selective anti-motility compound Active2 (termed antimotilin) in an early chronic H. pylori mouse model was significantly effective in reducing bacterial loads, both in the gastric corpus and the antrum. It also reduced quantitative molecular bacterial detection in the stomach at 2 weeks posttreatment almost below detection levels. This was in contrast to single antibiotic therapy using the well-established anti-H. pylori antibiotic metronidazole (4), which left no live bacterial counts at the same time point, but residual nonculturable bacteria that were still readily detectable by PCR and qPCR. This is a promising outcome for the novel anti-H. pylori principle, which encourages further investments into the therapeutic concept of antimotilin patho-blockers against H. pylori and other bacteria.

In conclusion, we have developed a novel type of antimotility screen specifically against the stomach pathogen H. pylori and identified promising patho-blocker compounds (antimotilins), one of which acted effectively on a steady-state early chronic H. pylori infection. The experiences in the animal model provide a promising outlook for a new kind of combination or even single therapy against H. pylori. Our results also indicate that antibiotic activity of compounds against H. pylori seems to impose a strong selective pressure and longer bacterial survival times in the stomach, which may, over the time of persistence, lead to increasing bacterial resistance development and higher probability of relapse. In contrast, the anti-motility compound led to a more rapid clearance of the bacteria from the stomach, which was clearly visible already at 2 weeks posttreatment, so far without recognizable bacterial resistance. In addition, the therapeutic dose of the patho-blocker compound did not alter the microbiota composition or richness of resident intestinal microbiota after 1 week of daily therapeutic intervention, which may indicate less prominent side effects. The effects *in vitro* seemed to be rather H. pylori-selective but not strain-specific. Further activities will be geared toward identifying the mode of action of Active2 and closely related compounds and a possible molecular target in the bacteria, and to address in more detail potential strain-specific effects and the possibility of resistance against the compound *in vivo*.

## MATERIALS AND METHODS

An extended version of Materials and Methods section is part of the supplemental material (Text S1).

### Bacterial strains and cultivation, including H. pylori.

H. pylori (strains N6, L7 for luciferase reporter; strains N6 (73), P12 (40), HP87P7 (24) for all other growth assays) was routinely cultured on blood agar plates supplemented with an antibiotic and antifungal combination under microaerobic conditions as described previously (74). For growth inhibition assays, H. pylori (strains N6, P12) was cultivated in broth culture (BHI broth, supplemented with 3% yeast extract and 5% horse serum) without antibiotic or antifungal supplement. Other bacterial strains used in the assays were Escherichia coli RP437 and Campylobacter jejuni 11186. See extended Materials and Methods for details as well as culture conditions for other bacterial species.

### H. pylori luminescence reporter strain, developed into a screening tool.

The H. pylori *flaA* promoter was transcriptionally fused with a luciferase operon (*luxAB*) from *Vibrio harveyii* in an H. pylori suicide plasmid (75). The *flaA* reporter fusion was recombined into the H. pylori chromosome and the resulting strain tested to be strongly luminescence-positive. Since transcriptional regulation of *flaA* is the culmination point of the flagellar hierarchy, the reporter principle will detect various inhibitory steps along the assembly and regulation of flagella, and provides a broadly selective principle for inhibitory effects, at the level of regulation, export, and assembly of the flagellar machinery. The primary flagellar reporter strain (N6 *flaA-luxAB*) activated luminescence to about 40,000 to 60,000 cps using luciferase substrate at an OD_600_ of 0.8 (mid-log-phase) in liquid culture. The signal-to-noise ratio of the assay is between 10^4^ and 10^5^. The Z-factor (76) was between 0.6 and 0.7, with a confidence interval of 95%. The reporter fusion was also introduced into a second H. pylori strain, L7 (77), with similar results. We validated the reporter strain using the compounds carbonyl cyanide m-chlorophenyl hydrazine (CCCP, inhibitor of membrane potential essential for flagellar motility), rotenone (inhibitor of complex I of the respiratory chain), and the antibiotic ampicillin (cell wall biosynthesis inhibitor). While the first two metabolic/respiratory chain inhibitors strongly inhibited the luminescence reporter activity, the cell wall antibiotic, which is highly effective against H. pylori, but does not directly affect motility or flagellar biosynthesis, did not alter the readings in the luciferase reporter assay. This clearly distinguishes the novel screening assay from a classical antibacterial screen.

As a counterscreen for viability and growth inhibition, a vitality assay based on respiratory activity quantitation with the redox dye tetrazolium violet was performed as described in the extended Materials and Methods in Supplemental Materials (Text S1) (78).

### General procedure of compound screening.

Four different libraries with compounds from commercial as well as academic sources were selected for screening in 96-well plates (Table 1). The final concentration of the compounds in the assays for initial screening was 10 μM (for MXL library) and 20 to 40 μM (LOPAC, ExNCL and SPECS libraries), depending on the available compound stock volumes. The final concentration of DMSO in the assays was always kept below 4%, since higher DMSO concentrations will interfere with metabolic functions of H. pylori and cause false-negative results.

### Luciferase/Bioluminescence assay.

H. pylori luminescence reporter strains were passaged on blood agar plates less than 22 h prior to the start of the experiment. Bacteria were harvested in brain heart infusion medium (BHI, 3% yeast extract, 5% horse serum) and adjusted to an OD_600_ of 0.8 in luciferase buffer (50 mM Na_2_HPO_4_, 2% BSA) at room temperature. 50 μl of the bacterial suspension were added per well of a 96-well microwell plate (nonbinding, Greiner Bio-One 655901).

Two μl of each of the compounds (in DMSO) were added to the sample wells. The same amount of pure, diluted DMSO (final concentration of a maximum of 4% per well) was added to the control wells. The microwell plate was incubated under microaerobic conditions at 37°C and with shaking at 175 rpm for 4 h.

Next, 10 μl of bacterial solution from each incubation well were transferred simultaneously to the wells of a white plate containing luciferase substrate and incubated on a microwell plate shaker for 10 s before the final measurements. For each measurement, at least four luciferase reporter positive-control wells (without inhibitory compound, with and without DMSO) and four to eight negative-control wells (background without bacteria) were run on the same day and under the same assay conditions in parallel.

### Calculation of the Z factor.

The Z factor was calculated as described by Zhang et al. (76).

### MIC, minimum bactericidal concentration (MBC).

In order to distinguish flagellar-effective compounds from classical antibiotics, H. pylori was subjected to MIC/MBC testing for selected compounds in liquid medium. See extended Materials and Methods in Supplemental Material (Text S1) for details.

MICs of known antibiotics (e.g., metronidazole) were also confirmed using etest strips (Liofilchem, Italy) on blood agar plates. The MIC for metronidazole for H. pylori wild-type strains (in particular the mouse-adapted strain HP87P7) was between 0.75 and 1.0 μg/mL as determined by etest. Hence the strains were fully metronidazole sensitive. For evaluation of the minimum bactericidal concentration (MBC) of E. coli or C. jejuni, bacteria from the respective MIC experiments in LB or MH broth were plated as streaks or in appropriate dilutions on LB agar. The LB agar plates were incubated in ambient atmosphere (E. coli) or under microaerobic conditions (C. jejuni) at 37°C overnight to detect growth. MIC/MBC measurements for selected compounds were also performed for Pseudomonas aeruginosa and Staphylococcus aureus in multi-well plates, using LB broth, at 37°C and ambient air over night.

### RNA-based assays to detect and quantitate compound effect on H. pylori transcript amounts.

For quantification of the effect of selected compounds on gene expression, pellets from 2 mL of bacterial growth (liquid BHI medium, final OD_600_ of 0.8) were subjected to RNA preparation and quantitative reverse transcriptase PCR (qPCR) as described previously (34, 38). The RNA was DNase I-treated, quality-controlled, reverse transcribed into cDNA, and *flaA* transcript was quantitated in qPCR using primers as specified in the results and figures. The qRT-PCR results were evaluated as fold change of *flaA* transcript in comparison to a noninhibited control. All specific transcript amounts were normalized to a H. pylori 16S control RT-PCR of each sample for comparison.

### Microscopic evaluation and motility tracking of H. pylori.

Tracking of H. pylori motility was performed in a microscope chamber (Olympus CELL-R system) set to an atmosphere of 37°C, 5% carbon dioxide and 50% humidity. See extended Materials and Methods in supplemental materials for details of media and experimental procedures. Compounds were added to the flasks, followed by gentle mixing, and motility of the bacteria was visually observed after 0, 15 min and 120 min of incubation. Movies were recorded for tracking with *Cell^R^* software (Olympus) after 15 min of incubation with the compounds. At least 20 bacterial cells visible for approximately 100 frames per movie were tracked using the *Cell^R^* system and software (23, 74). Velocity, number of stops/reversals and track lengths for each bacterial cell were observed and enumerated, with each back-and-forth motion (reversal) of a cell being considered one stop and each single stop accompanied by direction change counted as one stop.

### Active compound pretesting for mouse toxicity (preparation of preclinical model).

The active compound Active2, which was selected to be tested in a preclinical model, was initially tested for mouse toxicity by Maximal Tolerated Dose (MTD) testing. The MTD testing (MTD Tox56000 protocol, Eurofins Panlabs) was performed according to international standards. Briefly, three mice were dosed with the compounds at each intended dose. Active2 compound was dosed orally, at 10 mg/kg/day, 30 mg/kg/day, or 60 mg/kg/day, using a formulation of compound in 5% DMSO, 2.5% carboxy-methyl-cellulose (CMC), in PBS, also at 125 μl per dose per animal, followed by observation for 72 h. Compound Active2, used for the proof-of principle *in vivo* administration to treat H. pylori-infected mice, was very well soluble in water and did not show any toxicity in the animals up to 60 mg/kg/day, which made it suitable to be used in a subsequent animal treatment study.

### H. pylori infection and therapeutic compound administration in the mouse.

Six-to 8 weeks’ old specific pathogen-free C57BL/6 mice of mixed gender were obtained from Charles River Laboratories. In week one of the experiment, H. pylori mouse-adapted strain HP87P7, which was pretested in several adaptation rounds by PCR and genomic sequencing, to confirm stability of genetic traits and *cagPAI* genes and functionality, was administered at 3 × 10^5^ bacteria per mouse per inoculum (100 μl) by intragastric gavage on 2 days with 1 day of break in between. HP87P7 is fully metronidazole sensitive as determined by etest (MIC of 1 μg/mL). The proof-of-principle treatment was started 2 weeks after the end of the inoculation week, assuming a stable, early chronic, infection at this time point. The mice were treated in separate groups (8–10 animals per group), as outlined in the results, with compound(s), antibiotic (metronidazole at 14.3 mg/kg/day), a combination of both, antibiotic and compound, (for concentrations, see Results), or mock-treated once daily over seven consecutive days by intragastric gavage. After the treatment week, the mice were kept for another 2 weeks under normal housing and sterile feeding conditions. The mice were necropsied in week 7, the stomach was opened along the longer curvature and divided in half and additionally by antrum and corpus region. Antrum and corpus tissue segments were weighed and homogenized in BHI broth, 2.5% yeast extract, separately, and homogenates were plated on blood agar plates at appropriate dilutions. Cfus of H. pylori were counted after up to 6 days of growth on the plates. All reisolates were also PCR-tested, and selected clones were genome-sequenced, to detect any changed alleles or loss of *cagPAI* functionality. The latter was not the case. The animal experiments were authorized under German federal law by the LAVES (Lower Saxony Government Authority). The mouse infection with H. pylori was persistent for more than 6 weeks (the total duration of the experiment) but did not cause any discernible pathological features in the mouse stomach in our model as assessed by experienced, board-certified mouse pathologists (ADG, OK).

### Histopathology analyses.

Gastric tissue specimens from all experimental groups were sampled, processed for histopathology and scored as described (79) by board-certified mouse pathologists (OK, ADG). The scoring system is outlined in the Supplemental Materials (Text S1).

### Primer-probe qPCR to quantitate H. pylori in stomach tissue.

We developed a primer-probe PCR on the basis of the H. pylori *cagL* gene to detect H. pylori DNA/genome copies in homogenates of stomach tissue. As stated above, under our study conditions, mouse-adapted H. pylori strain HP87P7 did not lose *cagPAI* functions or genes *in vivo*. We verified the presence of H. pylori in all mouse stomachs after study conclusion using semiquantitative PCR with housekeeping gene primers (e.g., *ureB* gene); however, *ureB* did not work well in our set-up for quantitative primer-probe PCR assays. The primer-probe PCR protocol is provided in the Supplemental Materials and Methods file (Text S1). Subsequently, uniform band-size qPCR products were band-purified for sequencing and subjected to Sanger sequencing, in order to control for the unique gene target and target sequence variation in the mouse biopsy specimens. The PCR product identity was confirmed to be *cagL* in all sequenced samples from the mouse biopsy specimens. The limit of detection of this assay was determined to be about five genome copies as determined by serial DNA dilutions.

### Microbiota amplicon sequencing and analysis from mouse feces.

Lower bowel microbiota analysis was performed from fecal pellets collected from each animal both at the beginning (after the acclimatization period, a) and shortly before the end of the treatment experiment, b. 16S rRNA amplicon sequencing was performed for the identification of microbiota composition. The preparation of total DNA, 16S amplicon library preparations, microbiota sequencing (Illumina MiSeq Sequencer) and final bioinformatics analysis were done using Illumina Nextera XT chemistry and the bacterial 16S rDNA v3-v4 region-specific primers for 16S amplicon generation as previously described (80) (see Supplemental Materials and Methods [Text S1] for details). Briefly, paired sequencing reads obtained from 16S amplicons on a MiSeq instrument (Illumina) were collected, analyzed and searched for various bacterial taxonomic groups, down to genus or species level, using a standardized software pipeline comparing against several appropriate bacterial 16S databases. Principal coordinates analysis (PCoA) on OTU classifications was used for the visualization of beta-diversity. Beta-diversity analysis was performed on subsampled data using Bray-Curtis coefficient. PCoA (beta diversity) and alpha diversity (Shannon-Wiener index) were calculated using mothur version 1.39.5. Significance of overall diversity was calculated using AMOVA.

### Cytokine analysis from mouse blood.

Before the infection (controls, from a few selected mice only) and at the end of the treatment experiment, mouse blood was taken from the facial vein of each animal (approximately 30 to 100 μl per mouse). The blood plasma was subsequently separated from cells using separation centrifugation devices (Sarstedt Microvette, lithium-heparin). Plasma was diluted in Bio-Rad Bioplex assay buffer to one-fourth of the initial concentration and measured in the 23-Plex BioPlex bead-based multiplex cytokine assay (Bio-Rad number m60009rdpd) according to the manufacturer’s instructions and using the provided standards. Each sample was measured in a total volume of 50 μl in duplicates and >50 beads per analyte, and sample content of all included 23 cytokines were measured [pg/mL].

### Western blot and protein detection.

Details of protein analyses (SDS-PAGE, Western blot, antibodies) are provided in the extended Materials and Methods as supplemental material (Text S1).

10.1128/mbio.03755-21.1 TEXT S1 Supplemental materials and methods. Download Text S1, PDF file, 0.2 MB. Copyright © 2022 Suerbaum et al. 2022 Suerbaum et al. https://creativecommons.org/licenses/by/4.0/ This content is distributed under the terms of the Creative Commons Attribution 4.0 International license.
