# Correction for Suerbaum et al., “Identification of Antimotilins, Novel Inhibitors of Helicobacter pylori Flagellar Motility That Inhibit Stomach Colonization in a Mouse Model”

**DOI:** 10.1128/mbio.02301-22

**Published:** 2022-08-25

**Authors:** Sebastian Suerbaum, Nina Coombs, Lubna Patel, Dimitri Pscheniza, Katharina Rox, Christine Falk, Achim D. Gruber, Olivia Kershaw, Patrick Chhatwal, Mark Brönstrup, Ursula Bilitewski, Christine Josenhans

**Affiliations:** a Institute for Medical Microbiology and Hospital Epidemiology, Hannover Medical School, Hannover, Germany; b Max von Pettenkofer Institute for Hygiene and Medical Microbiology, Munich, Germany; c Department of Chemical Biology, Helmholtz Centre for Infection Research, Braunschweig, Germany; d Institute of Transplant Immunology, Hannover Medical School, Hannover, Germany; e Institute of Veterinary Pathology, Free University Berlin, Berlin, Germany; f German Center for Infection Research (DZIF), partner site Hannover-Braunschweig, Hannover, Germany; g German Center for Infection Research (DZIF), partner site Munich, Munich, Germany

## AUTHOR CORRECTION

Volume 13, no. 2, e03755-21, https://doi.org/10.1128/mBio.03755-21. One of the sources of funding for this study was not listed in the Acknowledgments section. The corrected Acknowledgments section should appear as shown below.

