# Supplementary material for: Identification of Antimotilins, Novel Inhibitors of Helicobacter pylori Flagellar Motility That Inhibit Stomach Colonization in a Mouse Model
Source: mBio. 2022 Mar 1;13(2):e03755-21. doi: 10.1128/mbio.03755-21 (PMC8941896; doi:10.1128/mbio.03755-21)
Supplement: TABLE S1 [file mbio.03755-21-st001.pdf]

H. pylori N6

Compound	Luminescence in first screen	IC_{50} luc	IC_{50} vit	MIC	MBC
Tyrphostin AG 879	18 %	0.34 µg/ml, 1.1 µM	0.03 µg/ml, 0.09 µM	1 µg/ml, 3.2 µM	4 µg/ml, 12.6 µM
L-750,667-trihydrochloride	5 %	0.87 µg/ml, 1.6 µM / 0.9 µg/ml, 1.7 µM	0.58 µg/ml, 1.1 µM	1 µg/ml, 1.9 µM	> 8 µg/ml, 15.2 µM
Calmidazoliumchloride	20 %	2.13 µg/ml, 3.1 µM / 2.7 µg/ml, 3.9 µM	0.13 µg/ml, 0.19 µM / 0.16 µg/ml, 0.23 µM	2 µg/ml, 2.9 µM	4 µg/ml, 5.8 µM
Z-L-Phe-chloromethylketone (ZPCK)	12 %	3.8 µg/ml, 11.5 µM / 4 µg/ml, 12.1 µM	5.4 µg/ml, 16.3 µM	0.5 µg/ml, 1.5 µM	4 µg/ml, 12.1 µM
Ebselen	15 %	2.6 µg/ml, 9.5 µM	2.9 µg/ml, 10.6 µM	4 µg/ml, 14.6 µM	4 µg/ml, 14.6 µM
SP600125	9 %	3.7 µg/ml, 16.8 µM	precipitated; n.d.	8 µg/ml, 36.3 µM	> 64 µg/ml
Calcimycine	9 %	0.04 µg/ml, 0.076 µM	0.01 µg/ml, 0.02 µM	0.25 µg/ml, 0.5 µM	0.5 µg/ml, 1 µM
NSC 95397	1 %	0.46 µg/ml, 1.48 µM	0.18 µg/ml, 0.58 µM	0.5 µg/ml, 1.6 µM	1 µg/ml, 3.2 µM
β-Lapachone	7 %	0.27 µg/ml, 1.11 µM	0.1 µg/ml, 0.4 µM	0.25 µg/ml, 1 µM	≥ 1 µg/ml, 4.1 µM
Flupirtinmaleate	3 %	not applicable	39.8 µg/ml, 94.7 µM	256 µg/ml, 609 µM	> 256 µg/ml
YC-1	1 %	0.22 µg/ml, 0.72 µM	6.13 µg/ml, 20.1 µM	4 µg/ml, 13.1 µM	16 µg/ml, 52.6 µM
Bay 11-7085	1%	0.7 µg/ml, 2.8 µM / 0.9 µg/ml, 3.6 µM	0.48 µg/ml, 1.9 µM	0.25 µg/ml, 1 µM	0.5 µg/ml, 2 µM
L-745,870-hydrochloride	22%	0.2 µg/ml, 0.55 µM	1.76 µg/ml, 4.8 µM	2 µg/ml, 5.5 µM	> 32 µg/ml, 88.1 µM
Chelerythrine-chloride	19%	2.28 µg/ml, 5.9 µM	6.97 µg/ml, 18.2 µM	8 µg/ml, 20.8 µM	16 µg/ml, 41.7 µM
Tyrphostin A9 / Malonoben	19%	0.04 µg/ml, 0.14 µM	0.01 µg/ml, 0.04 µM	0.125 µg/ml, 0.4 µM	4 µg/ml, 14.2 µM

H. pylori P12

Compound	MIC	MBC
Tyrphostin AG 879	2 µg/ml, 6.3 µM	8 µg/ml, 25.3 µM
L-750,667	1 µg/ml, 1.9 µM	> 256 µg/ml, 485.2 µM
Calmidazolium	4 µg/ml, 5,8 µM	16 µg/ml, 23.3 µM
Z-L-Phe-chloromethylketone (ZPCK)	0.125 µg/ml, 0.4 µM	8 µg/ml, 24.1 µM
Ebselen	4 µg/ml, 14.6 µM	8 µg/ml, 29.2 µM
SP600125	8 µg/ml, 36.3 µM	> 256 µg/ml, 1162.4 µM
Calcimycine	1 µg/ml, 1.9 µM	4 µg/ml, 7.6 µM
NSC 95397	0,5 µg/ml, 1.6 µM	1 µg/ml, 3.2 µM
β-Lapachone	0.125 µg/ml, 0.5 µM	0,25 µg/ml, 1 µM
Flupirtinmaleate	64 µg/ml, 152.2 µM	256 µg/ml, 609 µM
YC-1	16 µg/ml, 52.6 µM	32 µg/ml, 105.1 µM
Bay 11-7085	0.125 µg/ml, 0.5 µM	1 µg/ml, 4 µM
L-745,870	4 µg/ml, 11 µM	> 256 µg/ml, 704.7 µM
Chelerythrine-chloride	2 µg/ml, 5.2 µM	4 µg/ml, 10.4 µM
Tyrphostin A9 / Malonoben	0.125 µg/ml, 0.4 µM	> 0.5 µg/ml, 1.8 µM

Supplemental Table S1: specific compound characterization of selected compounds from LOPAC (repurposing) library with primary hit characteristics on the *H. pylori flaA* luciferase reporter strain. Selected compounds were tested in detail on two different *H. pylori* strains (N6, P12), demonstrating strain-independent reproducible compound effects. Since most of the compounds had strong effects on the vitality of *H. pylori*, P12 strain was exclusively tested for MIC and MBC values, revealing similar compund effects on both strains. P12 was slightly more sensitive than N6 in those assays. Bacteriostatic compounds in contrast to bactericidal compounds are distinguishable by high MBC values. The only compound with very little bacteriostatic or bactericidal effect, but strong motility inhibitory effect in this assay panel was flupirtinmaleate. All other compounds had strong antibacterial (“killing“) effects in vitro.
