# Supplementary material for: Identification of Antimotilins, Novel Inhibitors of Helicobacter pylori Flagellar Motility That Inhibit Stomach Colonization in a Mouse Model
Source: mBio. 2022 Mar 1;13(2):e03755-21. doi: 10.1128/mbio.03755-21 (PMC8941896; doi:10.1128/mbio.03755-21)
Supplement: TABLE S2 [file mbio.03755-21-st002.pdf]

C. jejuni 11168

Compound	MIC	MBC
Tyrphostin AG 879	> 256 µg/ml, 808.9 µM	> 256 µg/ml, 808.9 µM
L-750,667	> 256 µg/ml, 485.2 µM	> 256 µg/ml, 485.2 µM
Calmidazoliumchloride	4 µg/ml, 5.8 µM	8 µg/ml, 11.6 µM
Z-L-Phe-chloromethylketone (ZPCK)	32 µg/ml, 96.4 µM	128 µg/ml, 385.8 µM
Ebselen	16 µg/ml, 58.4 µM	16 µg/ml, 58.4 µM
SP600125	> 256 µg/ml, > 1162.4 µM	> 256 µg/ml, > 1162.4 µM
Calcimycin	> 256 µg/ml, 488.9 µM	> 256 µg/ml, 488.9 µM
NSC 95397	8 µg/ml, 25.8 µM	16 µg/ml, 51.5 µM
β-Lapachone	4 µg/ml, 16.5 µM	32 µg/ml, 132.1 µM
Flupirtinmaleate	256 µg/ml, 609 µM	> 256 µg/ml, 609 µM
YC-1	128 µg/ml, 420.6 µM	128 µg/ml, 420.6 µM
Bay 11-7085	4 µg/ml, 16 µM	8 µg/ml, 16 µM
L-745,870	> 256 µg/ml, 704.7 µM	> 256 µg/ml, 704.7 µM
Chelerythrine-chloride	4 µg/ml, 10.4 µM	32 µg/ml, 3.4 µM
Tyrphostin A9 / Malonoben	> 128 µg/ml, 453.3 µM	> 128 µg/ml, 453.3 µM

E. coli RP437

Compound	MIC	MBC
Tyrphostin AG 879	> 256 µg/ml, 808.9 µM	> 256 µg/ml, 808.9 µM
L-750,667	> 256 µg/ml, 485.2 µM	> 256 µg/ml, 4852 µM
Calmidazoliumchloride	32 µg/ml, 46.5 µM	> 256 µg/ml, 371.2 µM
Z-L-Phe-chloromethylketone (ZPCK)	> 256 µg/ml, 771.6 µM	> 256 µg/ml, 771.6 µM
Ebselen	32 µg/ml, 116.7 µM	64 µg/ml, 233.4 µM
SP600125	> 256 µg/ml, 1162.4 µM	> 256 µg/ml, 1162.4 µM
Calcimycin	> 256 µg/ml, 488.9 µM	> 256 µg/ml, 488.9 µM
NSC 95397	> 256 µg/ml, 824.8 µM	> 256 µg/ml, 824.8 µM
β-Lapachone	> 256 µg/ml, 1056.7 µM	> 256 µg/ml, 1056.7 µM
Flupirtinmaleate	> 256 µg/ml, 609 µM	> 256 µg/ml, 609 µM
YC-1	> 256 µg/ml, 841.2 µM	> 256 µg/ml, 841.2 µM
Bay 11-7085	> 256 µg/ml, 1026.8 µM	> 256 µg/ml, 1026.8 µM
L-745,870	> 256 µg/ml, 704.7 µM	> 256 µg/ml, 704.7 µM
Chelerythrine-chloride	> 256 µg/ml, 667 µM	> 256 µg/ml, 667 µM
Tyrphostin A9 / Malonoben	> 256 µg/ml, 906.6 µM	> 256 µg/ml, 906.6 µM

Supplemental Table S2: specific compound characterization of selected compounds from the LOPAC (repurposing) library with identified primary effects (all antibacterial effects except for flupirtinmaleate) on *H. pylori* were also tested for effects on *C. jejuni* strain 11168 and *E. coli* strain RP427. Some compounds also showed slight antibacterial effects on *C. jejuni*, indicating similar antibacterial targets in the two more closely related bacterial species. None of the compounds showed a clear antibacterial effect on *E. coli*. Those assays demonstrated that the novel (antibacterial) compound effects detected in this library were rather specific for *H. pylori* and possibly other *epsilonproteobacteria*.
