# Supplementary material for: Identification of Antimotilins, Novel Inhibitors of Helicobacter pylori Flagellar Motility That Inhibit Stomach Colonization in a Mouse Model
Source: mBio. 2022 Mar 1;13(2):e03755-21. doi: 10.1128/mbio.03755-21 (PMC8941896; doi:10.1128/mbio.03755-21)
Supplement: FIG S1 [file mbio.03755-21-sf001.pdf]

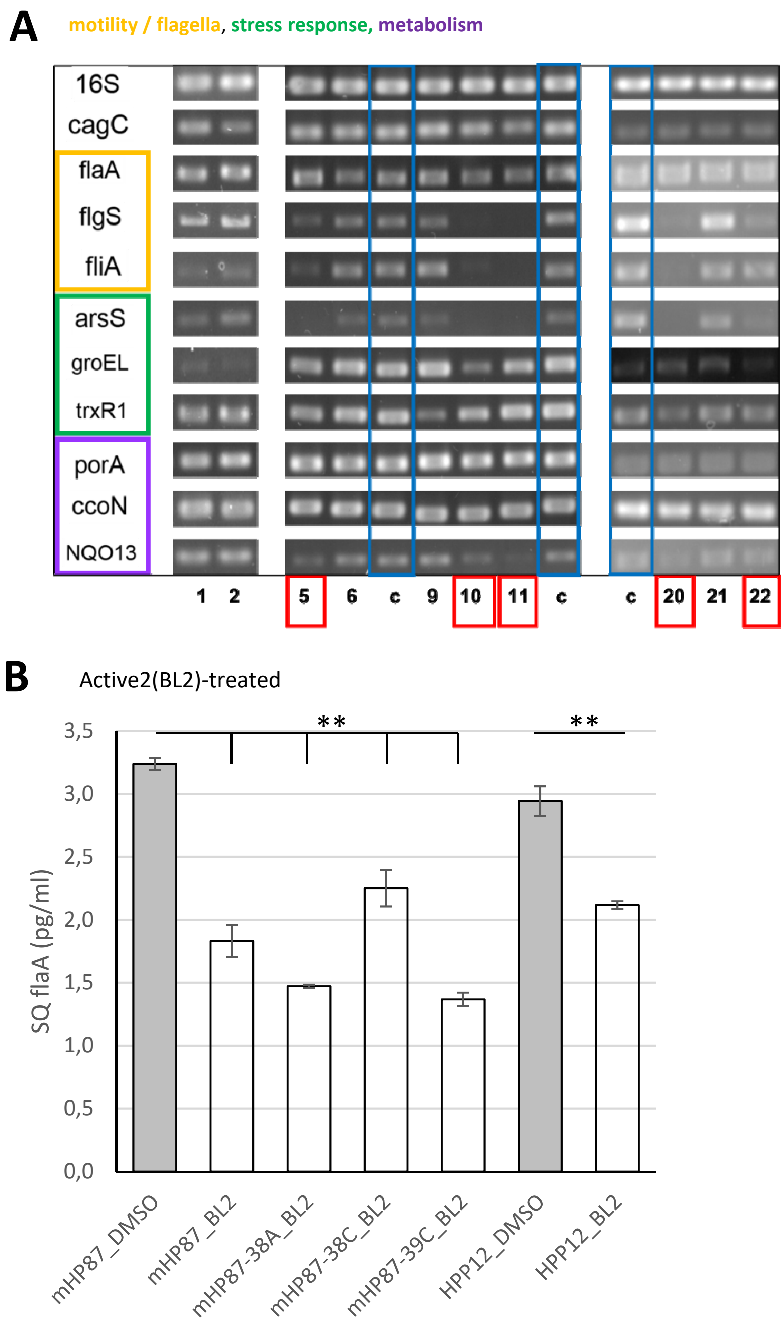

Fig S1: regulatory activity of primary small compound actives on specific and transcript amounts (RT-PCR). A) depicts semiquantitative (sq) RT-PCR results from RNA isolated from *H. pylori* N6 treated for 1 h in liquid culture of mid-log phase with 10 µg/ml of selected compounds (dissolved in DMSO) from the LOPAC library, containing repurposing compounds. Numbering of compounds in A) 1 -Calmidazolium, 2 -b-Lapachone (Active1), 5 –L745,870, 6 –NSC95397, 9 -paraquat, 10 –L-750,667, 11 –Tyrphostin AG879, 20 -ZPCK, 21 –SP600125, 22 –Bay11-7985; compounds are also listed in Supplemental Tables S1 and S2; compound 2 in A) is Active1= β-lapachone. Strongly active compounds are boxed in red color, which reduced transcript amounts of class 2 (*flgS*, *fliA, yellow*) and class 3 (*flaA, yellow*) flagellar genes as well as stress related (*arsS, green box*) and metabolic (*nqo13, violet box*) genes. In this case, the compounds had true antibacterial effects (see also Table S1 for MIC/MBC values). c are sq RT-PCRs performed on non-treated *H. pylori* N6 bacterial controls (DMSO only) incubated under the same conditions. Paraquat (9, not included in compound library) was used as an examplary set-up for a compound introducing oxidative stress. Gene abbreviations for RT-PCT: *cagC*, functionally important gene of the *cag* pathogenicity island; *flaA,* flagellin A subunit; *flgS*, flagellar regulator two component system (TCS), sensory protein; *fliA*, flagellar sigma factor σ28; *arsS* sensory domain of acid sensing TCS; *groEL*, heat shock protein, large subunit; *trxR1*, thioredoxin reductase; *porA*, pyruvate-ferredoxin oxidoreductase subunit; *ccoN*, N subunit of Cbb3 terminal oxidase; *nqo13*, subunit of complex I of respiratory chain; **B)** quantitative RT-PCR (qRT-PCR) results from RNAs collected from Active2(BL2) treatment in vitro (at 20 µg/ml for 4 h in liquid culture) of *H. pylori* mouse-adapted strain HP87 and HP87 bacteria reisolated from BL2-exposed mice from the treatment study (38A: mouse 38, antrum; 38C: mouse 38 corpus; 39C: mouse 39 corpus; mouse identities and group association see also Table 3 and Supplemental Fig. S3), and of independent *H. pylori* wild type strain P12. Strains N6 and L7 were characterized for Active2-effects on *flaA* by reporter assays. The HP87 reisolates from treated mice showed a similar response to Active2, indicating no primary acquired resistance to the compound effect on *flaA* during the treatment study. All *flaA* transcript amounts were normalized to 16S transcript. In this experiment under the short-term influence of Active2, no other gene transcripts of the above gene panel in A) were changed or downmodulated in the bacteria by Active2, coinciding with a lack of immediate growth- or stress-related Active2 effects on the bacteria, observed also in other assays for Active2.

Suerbaum et al., Fig S1
