# Supplementary material for: Identification of Antimotilins, Novel Inhibitors of Helicobacter pylori Flagellar Motility That Inhibit Stomach Colonization in a Mouse Model
Source: mBio. 2022 Mar 1;13(2):e03755-21. doi: 10.1128/mbio.03755-21 (PMC8941896; doi:10.1128/mbio.03755-21)
Supplement: FIG S2 [file mbio.03755-21-sf002.pdf]

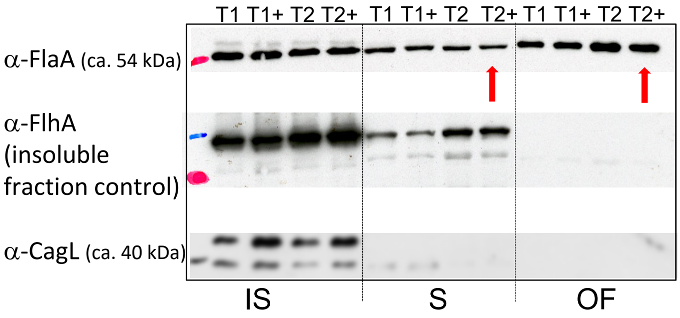

Fig S2: Western blot reveals reduction of flagellin protein FlaA upon incubation with active antimotilin compound Active2. Two different time points of an *H. pylori* growth curve in liquid BHI medium (3% yeast extract, 5% horse serum), T1 (early exponential, OD_{600} of about 0.4), and T2 (late exponential growth, OD_{600} of about 1.5) were monitored for differences in protein expression in the continuous presence (+) or absence of Active2 compound. Western immunoblots were performed on the insoluble (IS), soluble (S) and surface-associated (OF) fractions of the bacteria. Blots were developed with anti-FlaA, anti-FlhA and anti-CagL antisera as indicated. The FlaA blot reveals a significant reduction of main flagellin FlaA at time point T2 upon supplementation with Active2 compound, both in the soluble and surface-associated bacterial fractions (red arrows). FlhA serves as a fractionation control for the insoluble fraction. CagL, which was slightly increased by Active2 compound at both time points is shown as a second control for the insoluble fraction. Red marker band of prestained molecular mass marker runs at about 54 kDa.

Suerbaum et al., Fig S2
