# Supplementary material for: Identification of Antimotilins, Novel Inhibitors of Helicobacter pylori Flagellar Motility That Inhibit Stomach Colonization in a Mouse Model
Source: mBio. 2022 Mar 1;13(2):e03755-21. doi: 10.1128/mbio.03755-21 (PMC8941896; doi:10.1128/mbio.03755-21)
Supplement: TABLE S3 [file mbio.03755-21-st003.pdf]

group1a-group2a	Among	Within	Total
SS	0.176704	2.11979	2.2965
df	1	18	19
MS	0.176704	0.117766	
Fs:	1.50046		
p-value: 0.183			
group1b-group2b	Among	Within	Total
SS	0.0995619	1.91754	2.01711
df	1	18	19
MS	0.0995619	0.10653	
Fs:	0.934589		
p-value: 0.44			
group1b-group3b	Among	Within	Total
SS	0.124857	2.30859	2.43345
df	1	18	19
MS	0.124857	0.128255	
Fs:	0.973504		
p-value: 0.423			
group1b-group4b	Among	Within	Total
SS	0.0856737	2.17826	2.26393
df	1	18	19
MS	0.0856737	0.121014	
Fs:	0.707962		
p-value: 0.708			
group1b-group5b	Among	Within	Total
SS	0.113696	1.97407	2.08777
df	1	16	17
MS	0.113696	0.12338	
Fs:	0.921516		
p-value: 0.441			
group1b-group6b	Among	Within	Total
SS	0.223538	2.14301	2.36654
df	1	16	17
MS	0.223538	0.133938	
Fs:	1.66896		
p-value: 0.119			
group2b-group3b	Among	Within	Total
SS	0.0539685	2.04744	2.10141
df	1	18	19
MS	0.0539685	0.113747	
Fs:	0.474463		
p-value: 0.877			
group2b-group4b	Among	Within	Total
SS	0.236564	1.91711	2.15367
df	1	18	19
MS	0.236564	0.106506	
Fs:	2.22114		
p-value: 0.043			
group2b-group5b	Among	Within	Total
SS	0.0727183	1.71292	1.78564
df	1	16	17
MS	0.0727183	0.107057	
Fs:	0.679246		
p-value: 0.646			
group2b-group6b	Among	Within	Total
SS	0.289721	1.88185	2.17157
df	1	16	17
MS	0.289721	0.117616	
Fs:	2.46328		
p-value: 0.033			
group3b-group4b	Among	Within	Total
SS	0.275136	2.30815	2.58329
df	1	18	19
MS	0.275136	0.128231	
Fs:	2.14563		
p-value: 0.056			
group3b-group5b	Among	Within	Total
SS	0.0737357	2.10397	2.1777
df	1	16	17
MS	0.0737357	0.131498	
Fs:	0.560737		
p-value: 0.779			
group3b-group6b	Among	Within	Total
SS	0.296059	2.2729	2.56896
df	1	16	17
MS	0.296059	0.142056	
Fs:	2.08409		
p-value: 0.082			
group4b-group5b	Among	Within	Total
SS	0.217601	1.97363	2.19124
df	1	16	17
MS	0.217601	0.123352	
Fs:	1.76406		
p-value: 0.097			
group4b-group6b	Among	Within	Total
SS	0.148816	2.14257	2.29138
df	1	16	17
MS	0.148816	0.133911	
Fs:	1.11131		
p-value: 0.324			
group5b-group6b	Among	Within	Total
SS	0.222487	1.93838	2.16087
df	1	14	15
MS	0.222487	0.138456	
Fs:	1.60692		
p-value: 0.165			

Supplemental Table S3: statistics using AMOVA (Bray-Curtis) of 16S amplicon-based microbiota analyses (beta-diversity) of fecal pellets from Active2-treated mice and controls. The statistical analyses indicate no significant difference of the intestinal commensal microbiota composition (beta-diversity) between groups before or after the experimental course. Animal groups 1 through 6 are numbered as outlined in Table 3; additional group designation a: fecal microbiota before start of experiment after acclimatization period; additional group designation b: fecal microbiota after experimental course. P values of the respective comparisons are shaded in blue (non-significantly different) or light orange (trend towards significance). Differences between all mouse groups before the experiments (a) were also not significant; only one pairwise comparison between groups 1a and 6a as one representative comparison is listed for the pre-experimental status.

Suerbaum et al., Table S3
