# Supplementary material for: Identification of Antimotilins, Novel Inhibitors of Helicobacter pylori Flagellar Motility That Inhibit Stomach Colonization in a Mouse Model
Source: mBio. 2022 Mar 1;13(2):e03755-21. doi: 10.1128/mbio.03755-21 (PMC8941896; doi:10.1128/mbio.03755-21)
Supplement: FIG S3 [file mbio.03755-21-sf003.pdf]

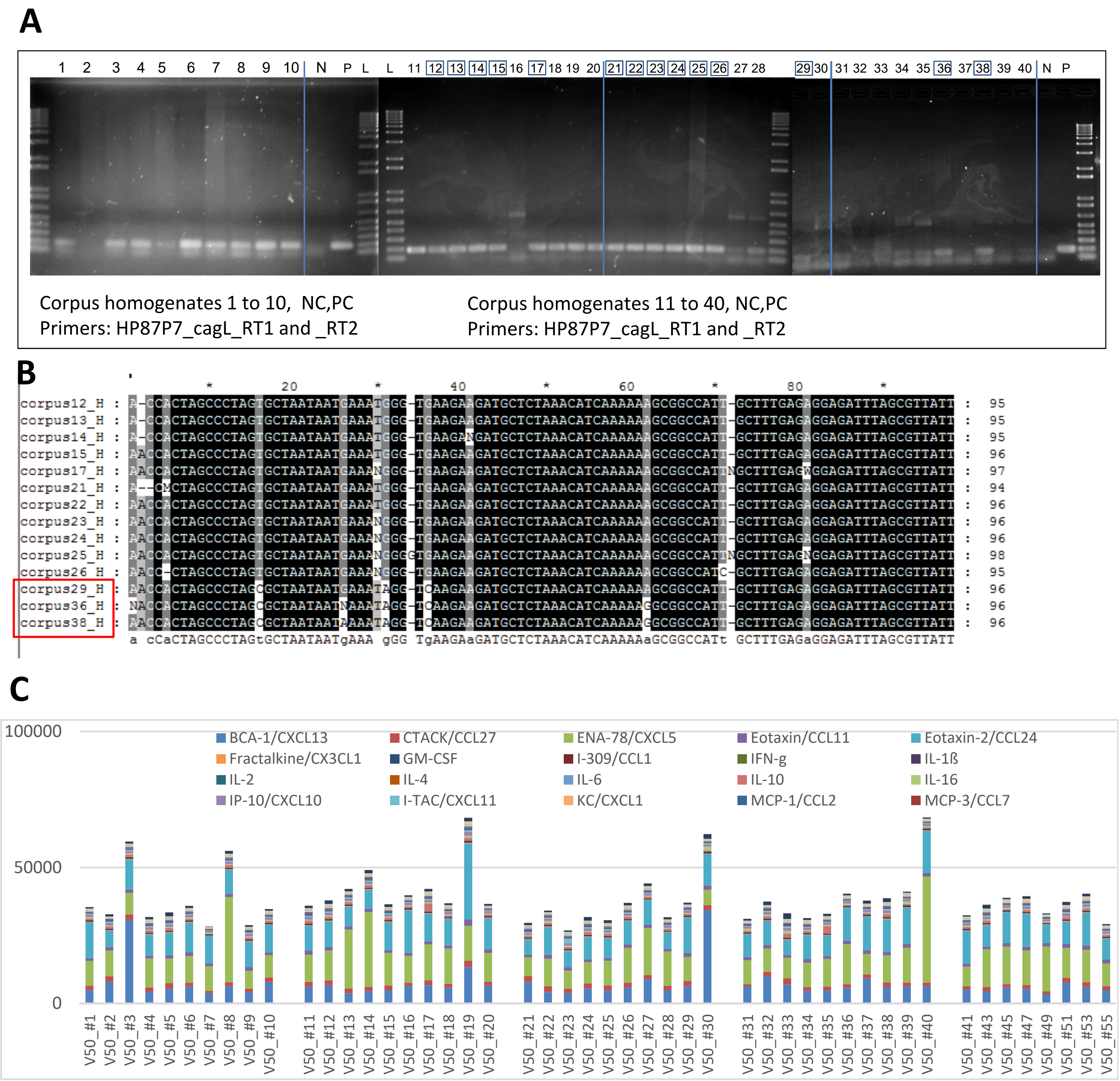

Fig S3: PCR identified strong residual bacterial load in a therapeutic *H. pylori* chronic infection model for the antibiotic metronidazole but not for the active pathoblocker/antimotilin compound BL2. No significant influence on plasma cytokine levels. A) a polymerase chain reaction for a partial nucleotide segment of the *H. pylori cagL* gene (Methods) was performed on stomach corpus homogenates from the chronic mouse infection and treatment experiment conducted with BL2. PCR results from mice grouped in four groups (only *H. pylori*-infected groups shown; groups separated by blue lines) (Table 3) are shown in the order of appearance. Group 1: mice 1 to 10; group 2: mice 11 to 20; group 3: mice 21 to 30; group 4 mice 31 to 40. Mouse numbers are shown above the PCR lanes. High-quality Sanger sequences obtained from *cagL* positive PCRs in the absence of positive culture (except for mouse 38, where two reisolate clones could be recovered) are boxed; respective Sanger sequence reads are shown in B). Nucleotide sequences from mice 29, 36 and 38 contain SNPs in comparison to the input strain (red box), while the sequences from the other reisolates and homogenate PCR bands do not contain SNPs. N = negative control without DNA; P = positive PCR control using genomic DNA of mouse-adapted *H. pylori* strain; L = nucleotide band size ladder - 1 kb ladder, Thermo Scientific.

C) No significant change of plasma cytokine levels (measured by BioRad Bioplex cytokine multiplex assay) was determined in chronic mouse infection model with or without therapeutic intervention with Active2. Groups of mice (six groups, 56 mice) are grouped as depicted in Table 3 and in the results. Results are shown in a cumulative manner in bar graphs, in pg/ml for each cytokine. Color labels for each cytokine are given in the figure. Of the mock-infected control groups 5 and 6, only every second mouse was tested as indicated. Differences between groups of mice were non-significant.

Suerbaum et al., Fig S3
