# Supplementary material for: Identification of Antimotilins, Novel Inhibitors of Helicobacter pylori Flagellar Motility That Inhibit Stomach Colonization in a Mouse Model
Source: mBio. 2022 Mar 1;13(2):e03755-21. doi: 10.1128/mbio.03755-21 (PMC8941896; doi:10.1128/mbio.03755-21)
Supplement: FIG S4 [file mbio.03755-21-sf004.pdf]

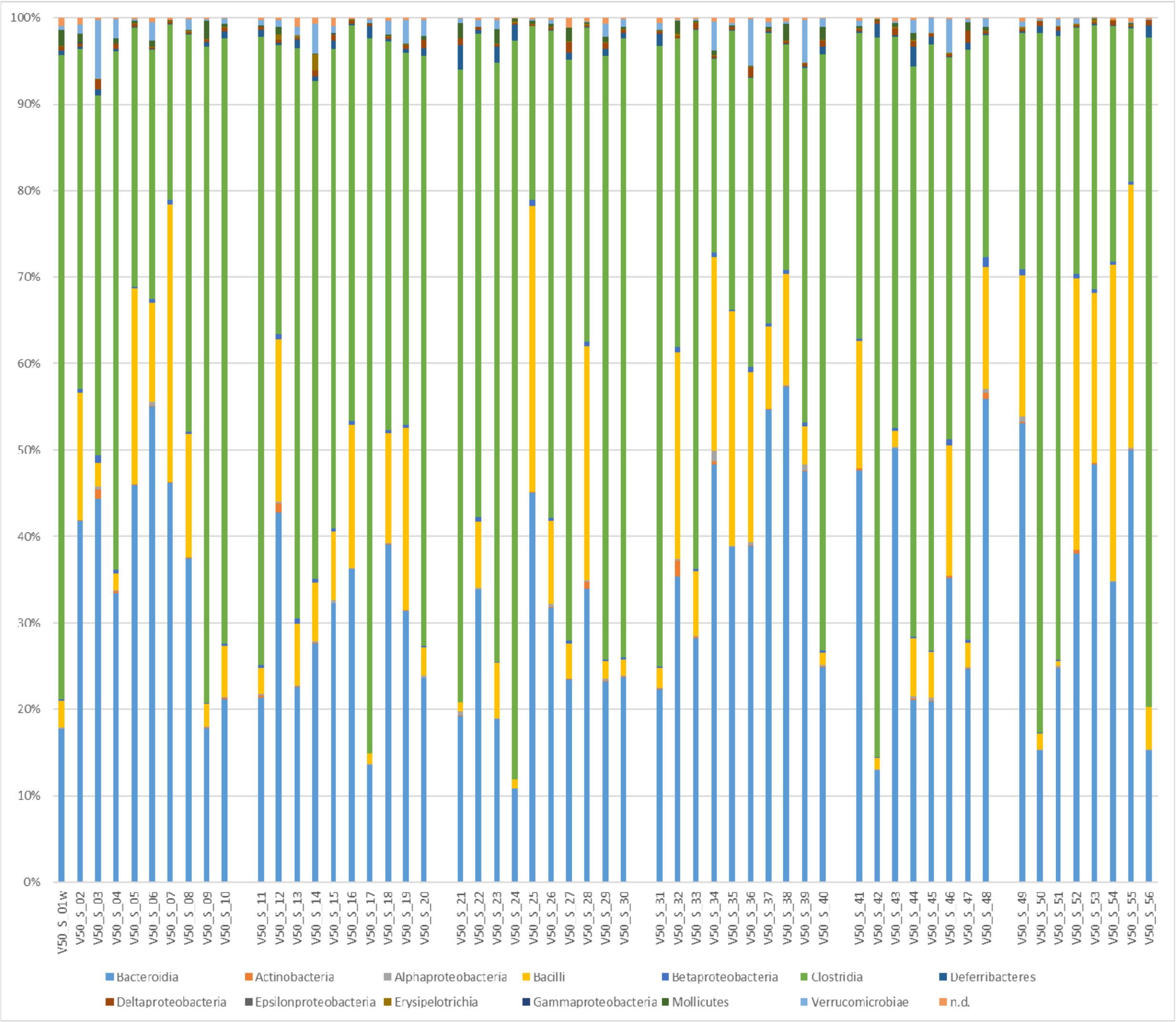

Fig S4: microbiota composition in the *H. pylori* chronic infection model post-treatment. All groups of mice were evaluated using 16S amplicon sequencing (Illumina MiSeq) from fecal pellets and subsequent OTU analysis, which were further grouped into taxonomic units (six groups, 56 mice, group details in Table 3 and in the Methods/Supplementary Methods). Shown here is the grouping into bacterial taxonomic classes, depicted as bar graphs for each mouse. Known classes of bacteria are shown with their respective color-coding in the legend below the figure; n.d. (orange) signify a small number of OTUs in the samples that could not be grouped into any of the known taxonomic classes. Main identified classes in all mice and mouse groups were the Bacteroidia, Clostridia and Bacilli. Differences in alpha diversity between mouse groups were not significant (Table S3).

Suerbaum et al., Fig S4
