# Supplementary material for: Identification of Antimotilins, Novel Inhibitors of Helicobacter pylori Flagellar Motility That Inhibit Stomach Colonization in a Mouse Model
Source: mBio. 2022 Mar 1;13(2):e03755-21. doi: 10.1128/mbio.03755-21 (PMC8941896; doi:10.1128/mbio.03755-21)
Supplement: TEXT S1 [file mbio.03755-21-s0001.pdf]

Suerbaum, Coombs, et al., Supplemental Materials, Methods and References

Identification of antimotilins, novel inhibitors of *Helicobacter pylori* flagellar motility that inhibit stomach colonization in a mouse model

Sebastian Suerbaum[1,2,6,7,§], Nina Coombs[1], Lubna Patel[2], Dimitri Pscheniza[1], Katharina Rox[3,6], Christine Falk[4], Achim D. Gruber[5], Olivia Kershaw[5], Patrick Chhatwal[1,6], Mark Brönstrup[3,6], Ursula Bilitewski[3,6], Christine Josenhans[1,2,6,7,§]

[1] Institute for Medical Microbiology and Hospital Epidemiology, Hannover Medical School, Hannover, Germany

[2] Max von Pettenkofer Institute for Hygiene and Medical Microbiology, Pettenkoferstrasse 9a, 80336 Munich, Germany

[3] Department of Chemical Biology, Helmholtz Centre for Infection Research, Braunschweig, Germany

[4] Institute of Transplant Immunology, Hannover Medical School, Hannover, Germany

[5] Institute of Veterinary Pathology, Free University Berlin, Berlin, Germany

[6] German Center for Infection Research (DZIF), partner site Hannover-Braunschweig, Hannover, Germany

[7] German Center for Infection Research (DZIF), partner site Munich, München, Germany

Supplemental Materials and Methods

Bacterial strains and cultivation, including *Helicobacter pylori.*

H. pylori (strains N6, L7 for luciferase reporter; strains N6 (Ferrero (1) et al., 1992), P12 (Fischer (2) et al., 2010), HP87 (3) for all other growth assays) was routinely cultured on blood agar plates (10% horse blood in Columbia blood agar base, Oxoid), supplemented with an antibiotic and antifungal combination under microaerobic conditions as described previously (4). For growth inhibition assays, *H. pylori* (strains N6, P12) was cultivated in broth culture (BHI broth, supplemented with 3% yeast extract and 5% horse serum), not supplemented with any antibiotics or antifungal agents. The microaerobic atmosphere required for growth was provided in air-tight incubation chambers (Scholzen Incubator) supplemented with a specific gas mixture (5% O_2, 10% CO_2, 85% N_2) or in anaerobic jars with oxygen-depleting gas generator bags (Anaerocult C, Merck). Other bacterial strains used in the assays were *Escherichia coli* RP437 and *Campylobacter jejuni* 11186. *E. coli* were routinely cultivated on LB plates, and liquid cultures for Minimum Inhibitory Concentration (MIC) assays were performed in LB broth medium under aerobic conditions. *C. jejuni* was grown on blood agar plates with 5% sheep blood, and liquid culture for antibacterial testing (MIC testing) was set up in BHI broth, supplemented with 3% yeast extract, under microaerobic conditions generated by CampyGen gas packs (Oxoid).

H. pylori luminescence reporter strain, developed into a screening tool.

The *H. pylori flaA* promoter was transcriptionally fused with a luciferase operon (*luxAB*) from *Vibrio harveyii* in an *H. pylori* suicide plasmid (5). The *flaA* reporter fusion was recombined into the *H. pylori* chromosome and the resulting strain tested to be strongly luminescence positive. Since transcriptional regulation of *flaA* is the culmination point of the flagellar hierarchy, the reporter principle will detect various inhibitory steps along the assembly and regulation of flagella, and thus a broadly selective principle for inhibitory effects, at the level of regulation, export, and assembly of the flagellar machinery. The primary flagellar reporter strain (N6 *flaA-luxAB*) activated luminescence to about 40,000 to 60,000 counts per second using luciferase substrate at an OD_{600} of 0.8 (mid-log phase) in liquid culture. The short incubation time of 4 h precludes growth of the slow-growing bacteria and allows for relatively fast screening. The negative control value of a non-reporter strain or reporter strain without luminescence substrate was close to zero. Hence, the signal-to-noise ratio of the assay is between 10^4 and 10^5. The Z-factor (6) determined for the assay with various bacterial inhibitors measured in quadruplicates or triplicates was routinely between 0.6 and 0.7, with a confidence interval of 95%. We also established the reporter fusion with similar results in a second *H. pylori* strain, L7 (7). We validated the reporter strain using the compounds carbonyl cyanide m-chlorophenyl hydrazine (CCCP) (inhibitor of membrane potential essential for flagellar motility), rotenone (inhibitor of complex I of the respiratory chain), and the antibiotic ampicillin (cell wall biosynthesis inhibitor). While the first two metabolic/respiratory chain inhibitors strongly inhibited the luminescence reporter activity, the cell wall antibiotic, which is highly effective against *H. pylori*, but does not directly affect motility or flagellar biosynthesis, did not alter the readings in the luciferase reporter assay. This clearly distinguishes the novel screening assay from a classical antibacterial screen. Phenamil (or

phenamil-methane-sulfonate), a known ion channel inhibitor, showed weak effects in the *flaA* luciferase screen (retained > 50% residual activity compared to non-inhibited control), while strongly inhibiting *H. pylori* growth. For the precise set-up of the luciferase measurements, see below.

As a counterscreen for viability and growth inhibition by any of the compounds, a vitality assay based on respiratory activity quantitation (based on the redox dye tetrazolium violet) was performed in 96-well plate format, with 100 µl of bacteria-reporter dye mix according to the manufacturers' instructions (reagents purchased from Biolog; see also (8). The reaction was incubated inside of a sealed PM Gas Bag (Biolog/PALL #3032) together with a CampyGen Compact pack (Oxoid), in order to create microaerobic conditions, at 37°C for approximately 30 h. Respiratory activity and redox dye activation in any sample is indicated by a color change from transparent to deep violet. Photometric measurement at the conclusion of the assay was performed in a TiterTek Multiskan multi-well reader at 492 nm.

General procedure of compound screening.

Four different libraries with compounds from commercial as well as academic sources were selected for screening (Table 1, Fig. 1). Compounds from the libraries were screened in sterile 96-well plates. Plate pipetting was performed semi-automatically using an EpMotion plate pipettor (Eppendorf). The compounds were first prepared as stocks in a uniform concentration and transferred in aliquots from the stock plate (384 wells or 96 wells) to the assay plate. The final concentration of the compounds in the assays for initial screening was 10 µM (for MXL library) or 20 µM to 40 µM (for LOPAC, ExNCL, and SPECS libraries) depending on the available volumes of each compound stock solution. The final concentration of DMSO in the assays was always kept below 4%, since higher DMSO concentrations will interfere with metabolic functions of *H. pylori* and cause false negative results.

Luciferase / Bioluminescence assay.

H. pylori luminescence reporter strains were passaged on blood agar plates less than 22 hours prior to the start of the experiment. The passage number for these assays was not higher than five to preserve the strain's properties and genetic stability. Luciferase reaction buffer was prepared as follows: 50 mM disodium phosphate (Na_2HPO_4) and 2 % BSA were dissolved in HPLC water, filter sterilized, aliquoted and frozen at – 20°C. Bacteria were harvested in brain-heart infusion medium (BHI, 3% yeast extract with 5% horse serum added) and adjusted to an OD_{600} of 0.8 at room temperature. 50 µl of the bacterial suspension were added per well of a 96 well microwell plate (non-binding, Greiner Bio-One 655901).

2 µl of each of the compounds (in DMSO) were added to the sample wells. The same amount of pure, diluted DMSO (final concentration of a maximum of 4% per well) was added to the control wells. The micro-well plate was incubated inside a sterile sealed PM Gas Bag (BIOLOG #3032) together with a CampyGen Compact pack (Oxoid) to generate microaerobic conditions at 37°C and with shaking at 175 rpm for 4 h.

Shortly before taking the measurements, the luciferase substrate was prepared by diluting the luminescence substrate decanal (10% stock, Sigma Aldrich) at a 1:2,000 dilution in luciferase reaction buffer. 60 µl of the substrate were added per well of a white 96 microwell plate (Thermo Scientific #236105) without the lid.

Next, 10 µl of bacterial solution from each inbubation well were transferred with the epMotion 96 (Eppendorf) simultaneously to the wells of the white plate containing the luciferase substrate and incubated on a microwell plate shaker for 10 s. The final measurements were recorded with a Perkin Elmer Victor 3/Wallac 1420 plate reader (luminometry, measurement time: 0.5 sec) or a Clariostar (BMG Labtech) plate reader and integrated to counts per sec. For the background measurement, 10 µl sterile medium containing 4% DMSO was added to the substrate wells. For each measurement, at least four luciferase reporter positive control wells (without inhibitory compound, with and without DMSO) and four to eight negative control wells (background without bacteria) were run on the same day and under the same assay conditions in parallel.

Calculation of the Z factor.

The Z factor was calculated as described by Zhang *et al*. (6)

$$Z = 1 - \frac{3\sigma_{\text{positive}} + 3\sigma_{\text{negative}}}{|\mu_{\text{positive}} - \mu_{\text{negative}}|}$$

σ - standard deviation, μ - mean, positive - positive controls, negative - negative controls

Minimum inhibitory concentration (MIC), minimum bactericidal concentration (MBC).

In order to distinguish flagellar-effective compounds from classical antibiotics, *H. pylori* was subjected to MIC/MBC testing for selected compounds. The bacteria were grown in liquid culture by harvesting bacteria from overnight-grown plates into brain-heart infusion medium (BHI, Oxoid) supplemented with 3% yeast extract and 5% horse serum, adjusted to an initial inoculum of OD_{600} = 0.05. 10 ml of this bacterial suspension were incubated as a pre-culture in a 50 ml Falcon tube with lid ajar inside of an air-tight incubation jar and a microaerobic atmosphere generated by Merck Anaerocult C gas generating packs at 37°C and shaking with 175 rpm overnight. On the following day, prior to the beginning of the experiment, the OD_{600} of the liquid culture was routinely below 1, in mid-exponential growth phase.

Next, 200 µl of the bacterial suspension were added per well of a 24-well-plate (Greiner Cellstar #662160) diluted to an initial OD_{600} of 0.07. Compounds (in DMSO) were added to the sample wells in duplicates in a serial dilution, starting at an upper limit of 256 µg/ml. Pure DMSO was added to all wells for a concentration of 2% DMSO per well. The 24-well plate (with lid) was incubated inside an incubation jar and under microaerobic atmosphere generated by Merck Anaerocult C sachets at 37°C, with shaking at 175 rpm for 24 h. For measurement, the duplicates were pooled after visual inspection, diluted 1:3 in medium and measured in plastic cuvettes at an OD of 600 nm. The detection limit of growth was defined as an OD_{600} of 0.05. Equal or lower values were considered as indicators of completely inhibited growth and counted as the Minimum Inhibitory Concentration (MIC). This MIC determination method can also be downscaled, further automated and measured in 96 or 384 well plates in a micro-well plate incubator equipped with a shaker-chamber for microaerobic bacterial incubation, while care has to be taken to shake the plate well.

MICs of known antibiotics (e.g. metronidazole) were also confirmed using Etest strips (Liofilchem, Italy) on blood agar plates. The MIC for metronidazole for *H. pylori* wild type strains (e.g. mouse-adapted strain HP87) was 0.75 to 1 µg/ml as determined by Etest. Thus the strains were fully sensitive against metronidazole.

For evaluation of the "minimum bactericidal concentration" (MBC) of test compounds, bacteria from the MIC experiment were streaked and plated at different serial dilutions on blood agar, including the lowest compound concentration where bacterial growth was still measurable by OD_{600}, and three compound concentration levels above (if possible). The blood agar plates were incubated at 37°C in an microaerobic atmosphere, either inside an incubation jar (Oxoid) with an Anaerocult C gas pack (Merck), or in an anaerobic cabinet aerated with a microaerobic gas mixture as outlined above (Scholzen).

For evaluation of the minimum bactericidal concentration (MBC) of *E. coli* or *C. jejuni*, bacteria from the respective MIC experiments in LB or MH broth were plated as streaks or in appropriate dilutions on LB agar. The LB agar plates were incubated in ambient atmosphere (*E. coli*) or under microaerobic conditions (*C. jejuni*) at 37°C overnight to detect growth. MIC/MBC measurements for selected compounds were also performed for *Pseudomonas aeruginosa* and *Staphylococcus aureus* clinical isolates in multi-well plates, using LB broth, at 37°C and ambient atmosphere overnight.

RNA-based assays to detect and quantitate compound effect on *H. pylori* transcript amounts.

H. pylori, inoculated to an initial OD_{600} of 0.4, was incubated in liquid broth medium (BHI supplemented with 2.5% yeast extract and 5% horse serum) for 4 h or up to an OD_{600} of 0.8, in the presence of respective compound(s) or DMSO only (positive control). Alternatively, in the case of compounds restricting the growth of bacteria, the bacteria were first pre-grown in broth culture up to an OD_{600} of 0.7 to 0.8, and were subsequently exposed to two log steps below MIC under sub-inhibitory concentrations of compounds or the respective concentration of the solvent DMSO for 1 h. Subsequently, the pellet of 2 ml of bacterial growth was subjected to RNA preparation and quantitative reverse transcriptase PCR (qPCR) as described previously (9, 10)(last reference includes our MIQE guidelines applied for qPCR). Briefly, the RNA was DNAseI-treated, quality controlled in an Agilent Bioanalyzer or Agilent Tape Station on RNA tapes, and reverse transcribed into cDNA, and *flaA* transcript quantitated in qPCR using primers flaA_RT1 and flaA_RT2, or other primer combinations as outlined in the results and figures, in comparison to the positive (non-inhibited) control. The qRT-PCR results were evaluated as fold-change of *flaA* transcript in comparison to a non-inhibited control. All specific transcript amounts were normalized to a *H. pylori* 16S control RT-PCR reaction of each sample for comparison.

Microscopical evaluation and motility tracking of motile *H. pylori.*

RPMI 1640 medium (Gibco), supplemented with 3% fetal bovine serum (Biochrom) was pre-warmed to 37°C. *Helicobacter pylori* (strain N6) was harvested in medium to a test OD_{600} of 0.033, distributed to cell culture flasks with filter lids (2 ml test volume each), and kept in a 37°C humidified incubation chamber at ambient atmosphere with 5 % carbon dioxide. The pre-incubation for equilibration of the bacterial culture was carried out for 15 min prior to the

beginning of the motility tracing. The microscope chamber (Olympus CELL-R system) was set to an atmosphere of 37°C, 5% carbon dioxide and 50% humidity. Compounds were added to the flasks, followed by gentle mixing. Motility of the bacteria was visually observed after 0, 15 min and 120 min of incubation. Movies were recorded for tracking with *Cell^R* software (Olympus) after 15 min of incubation with the compounds. At least 20 bacterial cells visible for approximately 100 frames per movie were tracked using the *Cell^R* system and software (4, 11). Velocity, number of stops/reversals and track lengths for each bacterial cell were observed and enumerated, with each back and forth motion (reversal) of a cell being considered as one stop and each single stop accompanied by direction change counted as one stop.

Active compound pretesting for mouse toxicity (preparation of preclinical therapeutic model).

The active compound Active2, which we selected to be tested in a preclinical model, was initially tested for mouse toxicity by Maximal Tolerated Dose (MTD) testing. The MTD testing (MTD Tox56000 protocol, Eurofins Panlabs) was performed according to international standards, as will be briefly outlined in the following: Three mice were dosed with the compounds at each intended dose. Active2 compound was dosed orally, at 10 mg/kg/day, 30 mg/kg/day, or 60 mg/kg/day, using a formulation of compound in 5% DMSO, 2.5% carboxy-methyl-cellulose (CMC), in PBS, also at 125 µl per dose per animal. The dosing in each case started with the lowest dose in the three animals, followed by observation for up to 72 h. Subsequently, the next higher dose was administered to a different set of three mice, etc.. Compound Active2, used for the proof-of principle in-vivo administration to treat *H. pylori*-infected mice, was very well water-soluble and did not show any toxicity in the animals up to 60 mg/kg/day, which made it suitable to use it in a subsequent animal treatment study.

H. pylori **infection and therapeutic compound administration in the mouse as a preclinical model for the antimotility therapeutic principle.**

6-8 weeks' old specific pathogen-free C57BL/6 mice of equally mixed gender were obtained from Charles River Laboratories. They were acclimatized to the new surroundings for one week before starting the assays. In week one of the experiment, *H. pylori* (mouse-adapted HP87P7, which was pretested in several adaptation rounds by PCR and genomic sequencing, not to lose genetic traits or *cagPAI* genes and functionality) was administered at $3x10^5$ bacteria per mouse per inoculum (100 µl) by intra-gastric gavage on two days with one day of break in between. Mouse-adapted HP87P7 is fully metronidazole-sensitive as determined by etest (MIC of 1 µg/ml). The proof-of-principle treatment was started two weeks after the end of the inoculation week, assuming a stable, early chronic infection at this time point. The mice were treated in separate groups (8-10 animals per group), as outlined in the results, with compound(s), antibiotic (metronidazole at 14.3 mg/kg/day, an established dosing in human anti-*H. pylori* therapy), a combination of both, antibiotic and compound, (for concentrations, see Results), or mock-treated once daily over seven consecutive days by intra-gastric gavage. After the treatment week, we kept the mice for another two weeks under normal housing and feeding conditions with sterilized chow and sterile water ad libitum. At the end of the assay period (week seven), the mice were necropsied, the stomach was opened along the longer

curvature and divided in half and additionally by antrum and corpus region. Antrum and corpus tissue segments were weighed and homogenized in BHI broth, 2.5% yeast extract, separately, and homogenates were plated on blood agar plates at appropriate dilutions. Cfus of *H. pylori* were counted after up to 6 days of growth on the plates. CFU counts were normalized to tissue weight. All reisolates were also PCR-tested and selected clones were whole genome-sequenced, to detect any changed alleles, or potential loss of *cagPAI* functionality. The latter was not the case. The animal experiments were authorized under German federal law by the LAVES (Lower Saxony Government Authority). The mouse infection with *H. pylori* was persistent for more than six weeks (the total duration of the experiment) but did not cause any discernible pathological features in the mouse stomach in our model as assessed by experienced, board-certified mouse pathologists (ADG, OK).

Mouse histopathology analyses.

Gastric tissue specimens from all experimental groups were sampled, processed for histopathology and scored as described (12). Briefly, tissues were immersion-fixed in 10% buffered formalin for 48 h, embedded in paraffin, sectioned at 4 µm thickness and stained with hematoxylin and eosin. Gastric inflammation was graded by board-certified mouse pathologists (OK, ADG), in a blinded fashion. The scoring system included the following criteria: 0, no significant lesions; 0.5, a slight abnormality such as a small focus of inflammatory infiltrate or extensive mucous metaplasia without inflammation; 1, a mild infiltrate of inflammatory cells usually along the base of the glands; 1.5, a mild infiltrate plus slight epithelial hyperplasia or extensive mucous cell metaplasia; 2, a larger focus of inflammation extending between glands and/or in submucosa; 2.5 inflammatory cells between glands and in the submucosa with mucous cell metaplasia and/or mild epithelial cell hyperplasia; 3, a patch of inflammation extending between glands toward the lumen and in the underlying submucosa often accompanied by moderate mucous cell metaplasia and mild to moderate epithelial hyperplasia; 3.5, more intense inflammation than 3.0 with marked epithelial hyperplasia; 4, an area of intense trans-mucosal inflammatory infiltrate which extends across the 10× field and obscures the normal architecture of the glands, usually accompanied by marked epithelial hyperplasia and extensive mucous cell metaplasia; 4.5, severe inflammation with focal ulceration of the mucosa; 5, extensive mucosal and submucosal inflammation with disruption of glandular architecture and ulceration. Ultimately, all mice in the experiment had negligible histopathological changes in the stomach, with scores ranging between of 0 and 0.5, and no significant differences between mouse groups.

Primer-probe qPCR to quantitate *H. pylori* in stomach tissue.

We developed a primer-probe PCR on the basis of the *H. pylori cagL* gene to detect *H. pylori* DNA/genome copies in homogenates of stomach tissue. As stated above, under our study conditions, mouse adapted *H. pylori* strain HP87P7 did not lose *cagPAI* functions or genes in vivo. We verified presence of *H. pylori* in all mouse stomachs after study conclusion using semi-quantitative PCR with housekeeping gene primers (e.g. using *ureB* gene primers); however *ureB* did not work well in our set-up for quantitative primer-probe PCR assays. *cagL* gene primers showed the most efficient and specific results of amplification in the mouse stomach homogenates, rendering those amenable to quantitative primer-probe PCR. The

primers used were: cagL_fw (FW), cagL_rv; probe primer labelled with FAM: cagL_probe (Probe). The protocol was run in a CFX96 BioRad qPCR machine with the following conditions: denaturation: 94 °C; annealing: 53°C; elongation: 68°C; number of cycles: 40. The final PCR products from the qPCR reactions were then also loaded on agarose gels for evaluation of DNA band size and uniformity. Subsequently, uniform band-size qPCR products were band-purified for sequencing and subjected to Sanger sequencing, in order to control for the unique gene target and target sequence variation in the mouse biopsies. Despite the fact that *H. pylori* cfus/reisolate growth were negative for some mice, in particular in mouse groups treated with metronidazole, we succeeded in raising a positive *cagL* PCR product in all of them, indicating remaining *H. pylori*, and also confirming that *cagL* gene or the *cagPAI* were not lost in this experiment. A selection of mouse reisolates was also tested in cell culture experiment for retainment of Cag type 4 secretion system functionality (IL-8 secretion assay), and all tested positively. The PCR product identity was ultimately indeed confirmed to be *cagL* in all sequenced samples from the mouse biopsies. The SQ results from the qPCR were normalized to individual biopsy tissue weight used for the homogenates in each individual tested animal. The limit of detection of this assay was determined to be about five genome copies as determined by serial DNA dilutions.

Microbiota amplicon sequencing and analysis from mouse feces.

Microbiota analysis was performed from fecal pellets collected from each mouse both at the beginning (after the acclimatization period, designation a in Table S3) and shortly before the end of the treatment experiment, designation b in Table S3. 16S rRNA amplicon sequencing was performed for the identification of microbiota composition. The preparation of total DNA, 16S amplicon library preparations, microbiota sequencing (Illumina MiSeq Sequencer) and final analysis were done using Illumina Nextera XT chemistry and the bacterial 16S rDNA v3-v4 region-specific primers for amplicon generation as previously described (13, 14).

Illumina forward and reverse reads were merged using the usearch software version 8.0 fastq_mergepairs command (15). Pairs were discarded if the overlap between the two sequences was shorter than 25 bp. Merged pairs were quality-filtered using the fastq_filter command to discard reads with more than 1.0 total expected errors. Primer sequences were removed using Tagcleaner v. 0.16 (16). No mismatches to 5' or 3' primers were permitted, and sequences with less than 330 bp were removed during the quality filtering.

Sequences were dereplicated using the Usearch derep_fulllength command and classified using the RDP classifier software version 2.12 Wang (17). Sequences with genus bootstrap >= 0.97 were identified and BLASTed against two databases (LTPs 128 and a modified version of the RDP 16S rRNA database release 11.5 that has been processed with Taxcollector 2.0 (18-20)), using the NCBI standalone BLAST software version 2.2.30 (21). In order to avoid memory constraints, the query dataset was split into subsets of 10 sequences each. BLAST results were limited to a maximum of 50 hit sequences for the LTPs database BLAST and 50,000 hit sequences for the RDP database, 97% minimum identity and a minimal BLAST e-value setting of 1E-50. Sequence classifications were retained if they agreed between both databases and the genus classification according to RDP classifier analysis was the same as for a LTPs database species sequence.

For further OTU classification, all further sequences were dereplicated and sorted by abundance using Usearch version 8.0 Edgar (15) derep_fulllength and sortbysize commands. Singletons were discarded. Clustering into operative taxonomic units (OTUs) was performed using the cluster_otus command. Chimeras were classified and removed using uchime with the "gold" reference database as implemented in the same Usearch version, OTUs were relabeled, and individual sequences were mapped to the OTU representative sequences at a minimum identity of 97%.

OTUs were then classified using the RDP classifier as described above and filtered to remove chloroplast sequences. OTUs identified as alphaproteobacteria were further tested to identify mitochondrial sequences using BLAST against the non-redundant Nr99 version of SILVA database 123 (20). OTUs with a class bootstrap value below 0.8 were also BLASTed against the non-redundant Nr99 version of SILVA database 123 at a minimum e-value of 1E-100 and 90% minimum identity. For each BLAST query, all hits with at least 95% identity covering of at least 400 bases match length were counted as good hits. For BLAST queries with at least 50 good hits, the hit with the best identity score (match length x percent identity) was retained and used for further analysis. Identified species and OTUs were then combined to a final dataset.

For further analysis with a subsampled dataset, we used mothur software version 1.39.5 (22). The dataset was subsampled to 38,042 sequences per sample corresponding to the sample with the lowest number of sequences. Alpha and beta diversity was calculated using mothur software version 1.39.5 (22). Metastats analysis (AMOVA) was performed using R software version 3.5.0 (23). Before performing the Metastats analysis, we removed features that were too rare to decide whether they are significantly overrepresented in one of the groups. Therefore, OTUs with less than 4 counts in 20% of the samples were removed. Final graphs of the diversity calculations were generated using Excel or Graphpad.

Cytokine analysis from mouse plasma.

Before the infection (controls, from a few selected mice only) and at the end of the treatment experiment, mouse blood aliquot was taken from the facial vein of each animal (approximately 30 to 100 µl per mouse). The blood plasma was subsequently separated from cells using separation centrifugation devices (Sarstedt Microvette, lithium-heparin). Plasma was diluted in BioRad Bioplex assay buffer to one-fourth of the initial concentration and measured in the 23-Plex BioPlex bead-based multiplex cytokine assay (BioRad #m60009rdpd) according to the manufacturer's instructions and using the provided standards. Each sample was measured in a total volume of 50 µl in duplicates and counting >50 beads per analyte, and sample concentrations of all included cytokines were quantitated [pg/ml].

Western blot and protein detection.

Protein amounts in bacterial fractions were determined by BCA assay (Thermo Fisher Scientific, Pierce) in appropriate dilutions. 10 µg of protein for each sample were separated on SDS gels (12.5% polyacrylamide in the separating gel portion). The gels were blotted onto nitrocellulose (Schleicher and Schuell, BA85 membrane) for 2 h at 350 mA. Membranes were

blocked using 5% blotting grade blocker (BioRad) in TBS buffer supplemented with 1% Tween 20 (TBS-T). Membranes were further incubated using selected specific rabbit antibodies directed against *H. pylori* flagellin and other *H. pylori* proteins used as loading and fractionation controls (FlhA, CagL). Primary antibody binding was detected by secondary antibodies coupled to horse-radish peroxidase (used at 1:10,000 dilution) and chemiluminescence substrate detection (Millipore/Immobilon high sensitivity chemiluminescent substrate).

Supplemental References

1. Ferrero RL, Cussac V, Courcoux P, Labigne A. 1992. Construction of isogenic urease-negative mutants of *Helicobacter pylori* by allelic exchange. J Bacteriol 174:4212-4217.
2. Fischer W, Windhager L, Rohrer S, Zeiller M, Karnholz A, Hoffmann R, Zimmer R, Haas R. 2010. Strain-specific genes of *Helicobacter pylori*: genome evolution driven by a novel type IV secretion system and genomic island transfer. Nucleic Acids Res 38:6089-6101.
3. Behrens W, Schweinitzer T, Bal J, Dorsch M, Bleich A, Kops F, Brenneke B, Didelot X, Suerbaum S, Josenhans C. 2013. Role of Energy Sensor TlpD of *Helicobacter pylori* in Gerbil Colonization and Genome Analyses after Adaptation in the Gerbil. Infect Immun 81:3534-3551.
4. Behrens W, Schweinitzer T, McMurry JL, Loewen PC, Buettner FF, Menz S, Josenhans C. 2016. Localisation and protein-protein interactions of the *Helicobacter pylori* taxis sensor TlpD and their connection to metabolic functions. Sci Rep 6:23582.
5. Niehus E, Ye F, Suerbaum S, Josenhans C. 2002. Growth phase-dependent and differential transcriptional control of flagellar genes in *Helicobacter pylori*. Microbiology 148:3827-3837.
6. Zhang JH, Chung TD, Oldenburg KR. 1999. A Simple Statistical Parameter for Use in Evaluation and Validation of High Throughput Screening Assays. J Biomol Screen 4:67-73.
7. Olbermann P, Josenhans C, Moodley Y, Uhr M, Stamer C, Vauterin M, Suerbaum S, Achtman M, Linz B. 2010. A global overview of the genetic and functional diversity in the *Helicobacter pylori cag* pathogenicity island. PLoS Genet 6:e1001069.
8. Gripp E, Hlahla D, Didelot X, Kops F, Maurischat S, Tedin K, Alter T, Ellerbroek L, Schreiber K, Schomburg D, Janssen T, Bartholomaus P, Hofreuter D, Woltemate S, Uhr M, Brenneke B, Gruning P, Gerlach G, Wieler L, Suerbaum S, Josenhans C. 2011. Closely related *Campylobacter jejuni* strains from different sources reveal a generalist rather than a specialist lifestyle. BMC Genomics 12:584. doi: 10.1186/1471-2164-12-584.:584-12.
9. Niehus E, Gressmann H, Ye F, Schlapbach R, Dehio M, Dehio C, Stack A, Meyer TF, Suerbaum S, Josenhans C. 2004. Genome-wide analysis of transcriptional hierarchy and feedback regulation in the flagellar system of *Helicobacter pylori*. Mol Microbiol 52:947-961.
10. Estibariz I, Overmann A, Ailloud F, Krebes J, Josenhans C, Suerbaum S. 2019. The core genome m5C methyltransferase JHP1050 (M.Hpy99III) plays an important role in orchestrating gene expression in *Helicobacter pylori*. Nucleic Acids Res doi:10.1093/nar/gky1307.
11. Schweinitzer T, Mizote T, Ishikawa N, Dudnik A, Inatsu S, Schreiber S, Suerbaum S, Aizawa S, Josenhans C. 2008. Functional characterization and mutagenesis of the proposed behavioral sensor TlpD of *Helicobacter pylori*. J Bacteriol 190:3244-55.
12. Garhart CA, Redline RW, Nedrud JG, Czinn SJ. 2002. Clearance of *Helicobacter pylori* Infection and Resolution of Postimmunization Gastritis in a Kinetic Study of Prophylactically Immunized Mice. Infect Immun 70:3529-38.
13. Takahashi S, Tomita J, Nishioka K, Hisada T, Nishijima M. 2014. Development of a prokaryotic universal primer for simultaneous analysis of Bacteria and Archaea using next-generation sequencing. PLoS One 9:e105592.
14. Yang I, Woltemate S, Piazuelo MB, Bravo LE, Yepez MC, Romero-Gallo J, Delgado AG, Wilson KT, Peek RM, Correa P, Josenhans C, Fox JG, Suerbaum S. 2016. Different gastric microbiota compositions in two human populations with high and low gastric cancer risk in Colombia. Sci Rep 6:18594.
15. Edgar RC. 2013. UPARSE: highly accurate OTU sequences from microbial amplicon reads. Nat Methods 10:996-8.
16. Schmieder R, Lim YW, Rohwer F, Edwards R. 2010. TagCleaner: Identification and removal of tag sequences from genomic and metagenomic datasets. BMC Bioinformatics 11:341.
17. Wang Q, Garrity GM, Tiedje JM, Cole JR. 2007. Naive Bayesian classifier for rapid assignment of rRNA sequences into the new bacterial taxonomy. Appl Environ Microbiol 73:5261-7.

18. Yarza P, Richter M, Peplies J, Euzeby J, Amann R, Schleifer KH, Ludwig W, Glockner FO, Rossello-Mora R. 2008. The All-Species Living Tree project: a 16S rRNA-based phylogenetic tree of all sequenced type strains. Syst Appl Microbiol 31:241-50.
19. Dias R, Xavier MG, Rossi FD, Neves MV, Lange TA, Giongo A, De Rose CA, Triplett EW. 2014. MPI-blastn and NCBI-TaxCollector: improving metagenomic analysis with high performance classification and wide taxonomic attachment. J Bioinform Comput Biol 12:1450013.
20. Yilmaz P, Parfrey LW, Yarza P, Gerken J, Pruesse E, Quast C, Schweer T, Peplies J, Ludwig W, Glockner FO. 2014. The SILVA and "All-species Living Tree Project (LTP)" taxonomic frameworks. Nucleic Acids Res 42:D643-8.
21. Camacho C, Coulouris G, Avagyan V, Ma N, Papadopoulos J, Bealer K, Madden TL. 2009. BLAST+: architecture and applications. BMC Bioinformatics 10:421.
22. Schloss PD, Westcott SL, Ryabin T, Hall JR, Hartmann M, Hollister EB, Lesniewski RA, Oakley BB, Parks DH, Robinson CJ, Sahl JW, Stres B, Thallinger GG, Van Horn DJ, Weber CF. 2009. Introducing mothur: open-source, platform-independent, community-supported software for describing and comparing microbial communities. Appl Environ Microbiol 75:7537-41.
23. White JR, Nagarajan N, Pop M. 2009. Statistical methods for detecting differentially abundant features in clinical metagenomic samples. PLoS Comput Biol 5:e1000352.
